# Marriage and divorce during a pandemic: the impact of the COVID-19 pandemic on marital formation and dissolution in Mexico

**DOI:** 10.1007/s11150-023-09652-y

**Published:** 2023-04-11

**Authors:** Lauren Hoehn-Velasco, Jose Roberto Balmori de la Miyar, Adan Silverio-Murillo, Sherajum Monira Farin

**Affiliations:** 1 Department of Economics, Andrew Young School of Policy Studies, Georgia State University, Atlanta, GA USA; 2 Business and Economics School, Universidad Anáhuac México, Mexico City, Mexico; 3 School of Government, Tecnologico de Monterrey, Monterrey, Mexico

**Keywords:** COVID-19, Pandemic, Divorce rates, Marriage rates, Mexico, J11, J12, J13

## Abstract

In this study, we consider the initial effects of the COVID-19 pandemic on family formation and dissolution. We use national microdata covering all marriages and divorces in Mexico, an event-study design and a difference-in-difference specification. Our findings indicate that over March through December of 2020, marriage rates declined by 54% and divorce rates by 43%. By the end of 2020, divorce rates recover back to baseline levels, but marriage rates remain 30% below the 2017–2019 baseline level. Overall, our findings suggest that marital dissolutions quickly recovered (6 months into the pandemic), but at the end of 2020, family formation remained at persistently lower levels.

## Introduction

Over 2020, the COVID-19 pandemic disrupted social activity throughout the world. One specific area of society presumably impacted by the pandemic is the formation and dissolution of marriages. Marriage, divorce, and fertility rates all follow pro-cyclical patterns, and the pandemic recession produced substantial income loss for Mexican households (Hoehn-Velasco, de la Miyar & Penglase, 2022). This income loss may cause households to postpone structural changes such as marriage and divorce (as is typical during recessions, Chowdhury (2013), Kondo (2016)).^1^

The unprecedented national stay-at-home order issued on March 23rd, 2020, induced substantial additional costs to household rearrangement, especially due to the closure of courts. The fear of infection and limitations on gatherings during the pandemic also potentially fundamentally changed union formation. The distinct aspects of the pandemic may more closely reflect the demographic consequences of a natural disaster rather than a typical recession. In some instances, natural disasters have increased marriage and divorce rates, but the findings in the literature are mixed.^2^ Thus, the overall impact of the pandemic on family formation and dissolution, and the duration of this social disruption remains unclear.

In this study, we consider the initial effects of the COVID-19 pandemic on family formation and dissolution, focusing on the impacts over the first wave of the pandemic (during 2020). We use national administrative marriage and divorce records from the Instituto Nacional de Estadística y Geografía (INEGI). These microdata cover all divorces and marriages in Mexico over our study period, 2017–2020. The records include a rich set of couple-specific characteristics, as well as the type of marriage/divorce. For the divorce records, we also know the specific cause of divorce, which member of the couple filed for the divorce, and the length of the marriage (prior to divorce).

We consider the impact of the pandemic on marriage and divorce using aggregate state-level marriage and divorce rates. To calculate the rates, we combine the aggregated microdata with population counts for state-level inhabitants aged 15 and over.^3^ Our final dataset includes a monthly state-level series of marriage and divorce rates from 2017 to 2020. In addition to the marriage (and divorce) rates, our data allows us to consider the characteristics of marriages (and divorces) as a percentage of total marriages (and divorces). Changing to a percentage specification enables us to assess whether there was a short-term compositional shift in the attributes of couples during the pandemic.

Using this state-level monthly series, we analyze the impact of the pandemic using an event-study design and a difference-in-differences specification. For our difference-in-differences specification, because we do not have untreated units, we use year-over-year changes in the marriage and divorce rates. Thus, 2020 represents our treatment year, which we compare to the intertemporal variation in the control years 2017–2019. Our second source of variation occurs within the treatment and control years, where we compare pre-pandemic months (January–February) to post-pandemic months (March and onward).^4^,^5^ Since the latest available marriage and divorce data in Mexico only covers the first wave of the COVID-19 pandemic; our conclusions are limited to the initial impacts of the pandemic.

Our findings show a sharp decline in both marriage and divorce rates over the beginning of the pandemic, especially during the stay-at-home order. After the stay-at-home order ends, both marriage and divorce rates begin to recover. Divorce rates recover faster than marriage rates and are back to baseline levels by September 2020. Marriage rates recover partially but fail to reach 2017–2019 levels by the end of 2020. On average, from March through December 2020, divorce rates declined by 43%, and marriage rates declined by 54%. These findings indicate that, during the first wave of the COVID-19 pandemic, couples continued to end marriages through divorce. However, new marriages were still postponed at the end of 2020 (if not permanently reduced).

Then, we assess the specific characteristics of marriages and divorces during the pandemic, considering whether there were compositional adjustments. The findings show that marriages and divorces skew younger during the pandemic, suggesting that the youngest individuals are the least likely to defer plans during the pandemic. Divorcing wives are more likely to be employed and less likely to initiate divorce. Instead, couples jointly file for divorce, making use of administrative divorce rather than going through a judicial process. Educational attainment of new marriages and divorces also adjusts. Marriages shift towards couples with higher levels of education (above high school), while divorces shift towards those with primary education or less.

These findings fit into a small but growing body of literature analyzing the effects of the COVID-19 pandemic on marriages and divorces. In particular, studies in Denmark (Fallesen, 2021), Japan (Ghaznavi et al., 2022, Komura & Ogawa, 2022), South Korea (Kim & Kim, 2021), and the United States (Manning & Payne, 2021, Wagner, Choi & Cohen, 2020, Westrick-Payne, Manning & Carlson, 2022) all show a reduction in both new divorces (ranging from 3% to 27%) and new marriages (ranging from 10% to 14%) during the pandemic (Fallesen, 2021, Kim & Kim, 2021, Komura & Ogawa, 2022, Westrick-Payne et al., 2022). Our study fills several remaining gaps in this literature by considering a middle-income setting, documenting the dynamic effects of the pandemic, and considering the compositional shifts of new marriages and divorces during the pandemic.

Most notably, our study is among the first to provide *causal* evidence of the short-term impact of the pandemic in the context of a middle-income economy. Our findings indicate a larger decline in new marriages and divorces during the COVID-19 pandemic in Mexico, suggesting that demographic responses in the middle-income setting may vary from high-income counterparts. Mexico, and similar countries, may have had more limited access to social safety nets as the governmental response was relatively weak (IMF, 2021). These structural differences make the potential impacts of the pandemic in the middle-income setting more severe than in high-income counterparts.

## Literature

### Related studies and contribution

The majority of the existing literature studying the impact of the COVID-19 pandemic on marriage and divorce rates focuses on high-income settings. Fallesen (2021) use a monthly series of divorce filings in Denmark, and show that the filing rate was 7% lower during the pandemic. In Japan, Komura and Ogawa (2022) find that the pandemic-induced nationwide state of emergency caused the number of marriages and divorces (per 1000 population) to decline by 10% and 27%, respectively.^6^ In South Korea, Kim and Kim (2021) use administrative data and find a 10–14% reduction in marriage rates and a 3–7% reduction in divorce rates. Finally, in the United States, Westrick-Payne et al. (2022) consider data on marriages from 20 states and divorces from 35 states and find an 11% shortfall in marriages and a 12% shortfall in divorces.^7^

Our findings in Mexico, a middle-income setting, suggest a similar decline in new marriages and new divorces. However, Mexico’s decline in new marriages (54%) and divorces (43%) is substantial when compared with related studies in high-income countries, which range from a reduction in new divorces by 3% to 27% and new marriages by 10% to 14% (Fallesen, 2021, Kim & Kim, 2021, Komura & Ogawa, 2022, Westrick-Payne et al., 2022). The larger reduction in marriage and divorce rates in Mexico may be due to several factors, the most notable being the limited supply of vaccines, the weak economic relief response of the Mexican government, as well as limited administrative capacity of the government.^8^

A second contribution of the present study is to consider disaggregate data and the dynamic impacts of the pandemic. The majority of studies to date have focused on the overall aggregate impacts of the pandemic (Fallesen, 2021, Kim & Kim, 2021, Komura & Ogawa, 2022, Westrick-Payne et al., 2022). However, these aggregate numbers fail to demonstrate the dynamic effects of the pandemic as well as the compositional adjustments of new marriages and divorces during the pandemic (Komura & Ogawa, 2022). In the present study, we show that, in Mexico, by the end of 2020, marriage decisions were still being postponed (if not permanently prevented), but divorces returned to pre-pandemic levels.

Mexico’s data also allows us to consider the characteristics of new marriages and divorces before and during the pandemic, including age, education, and employment status. The rich microdata demonstrates the pandemic-induced compositional shifts in the attributes of couples opting for marriages or divorces. These compositional adjustments are important to consider, as they have implications for marital assortativeness and inequality across households (Breen & Salazar, 2011, Eika, Mogstad & Zafar, 2019, Greenwood, Guner, Kocharkov & Santos, 2014), and Mexico’s level of inequality is already higher than other OECD countries (Hoehn-Velasco & Penglase, 2021a). While we do not directly assess inequality or assortativeness in the present study, our findings suggest that new marriages shift towards the most educated, and new divorces shift towards the least educated. These results provide suggestive evidence of who-marries-(and divorces)-who during the pandemic, and opens avenues for future research into the effects of the pandemic on assortativeness and inequality in Mexico. Finally, our findings add to the broader literature focusing on the gendered impact of the COVID-19 pandemic, which could potentially affect outside options, especially for women.^9^

### Anticipated impacts of the pandemic

The far-reaching detrimental impacts of the COVID-19 pandemic affect couples’ marriage and divorce decisions through several channels. The economic, social, and psychological offshoots of the COVID-19 pandemic include unemployment (Schmid, Wörn, Hank, Sawatzki & Walper, 2021), loss of income (Hoehn-Velasco et al., 2022), overall uncertainty (Guetto, Vignoli & Bazzani, 2021), and emotional stress due to isolation (Luetke, Hensel, Herbenick & Rosenberg, 2020), which have the potential to shape couple dynamics.

First and foremost, the governmental restrictions through lockdowns and stay-at-home orders made it physically and logistically difficult to obtain a marriage or divorce certificate (Goldberg, Allen & Smith, 2021, Komura & Ogawa, 2022, Manning & Payne, 2021). The restrictions on social gatherings directly contributed to declines in marital formation by making wedding ceremonies unavailable (Kim & Kim, 2021), and pushed couples to postpone marriage (Testa, 2020). This channel is particularly applicable to an established couple willing to get married, though the fear of infection and limitations on gatherings and travel during the pandemic may have also disrupted relationship-building processes among short-term dating couples (Kim & Kim, 2021). For divorces, the implementation of stay-at-home orders potentially interfered with the launching and proceeding of divorce processes (Lebow, 2020).

Second, economic models of marital decision-making suggest that during economic downturns, the income effect due to stability of earnings may dominate the substitution effect arising from the higher opportunity cost of financial insecurity, single parenting, etc.^10^ Prospects of increased gains from marriage led by shared consumption and insurance against adverse shocks to earnings, income, or health (Shore, 2010, Stevenson & Wolfers, 2007) conceivably increase marital formation. By contrast, adverse changes in labor market conditions (i.e., wage and employment status) could lead to loss of gains from marriage, especially for men (Becker, 1973). This indicates a potential negative relationship between unemployment and marital formation, suggesting marriages are pro-cyclical (González-Val & Marcén, 2018, Schaller, 2013).

Similar to marriages, divorces are also affected by poor macroeconomic conditions (recessions) for several financial reasons. First, filing for the divorce itself is expensive in most cases. Thus, couples are more likely to postpone incurring this “cost of divorce” till their income improves (Adshade, 2012, Amato & Beattie, 2011, Chowdhury, 2013). Second, in a household with dual-earning partners, each spouse’s job acts as unemployment insurance for the other. This can be linked directly to Becker’s original model on marital instability, which views marriage as insurance against economic hardship (Becker, Landes & Michael, 1977). Thus, during recessions, higher unemployment, fear of imminent job loss, and the general decline in economic expectations could potentially lead individuals to delay divorce to keep access to all forms of financial security (Amato & Beattie, 2011, González-Val & Marcén, 2018, Hurd & Rohwedder, 2010). Lastly, the decline in divorces can be associated with deterioration in the housing market, or any asset market for that matter, which is generally the case during recessionary times (Chowdhury, 2013, Cohen, 2014, Farnham, Schmidt & Sevak, 2011).

In Mexico, 346,878 formal jobs were lost in only 24 days, from March 18 to April 6 (Montoya, Azuara & Rubio, 2020). Wages in Mexico also declined substantially over the initial months of the pandemic, with men and women similarly impacted (Hoehn-Velasco et al., 2022). Due to the economic hardship, a decline in both marriage and divorce rates may be expected, with potential persistent effects.^11^ The effects of the COVID-19 pandemic may be “dangerously unique” (Borio, 2020) and the scale of the COVID-19 economic downturn relative to past pandemics is unprecedented (Arthi & Parman, 2021, Borio, 2020, Rose, 2021). Thus the impacts of the economic downturn on family formation should be significant. This hardship likely increases conflicts among couples and potentially leads to an increased likelihood of marital dissolution (Hardie & Lucas, 2010, Masarik et al., 2016).

Third, another important potential mechanism is the pandemic-related emotional response to risk and uncertainty. Life-threatening events motivate people to take significant life-altering actions, especially in their close relationships (Cohan & Cole, 2002). The distinct aspects of the pandemic may compare most closely with those of a natural disaster (rather than a typical recession). Economic crises and natural disasters deemed as major “stressors” are likely to affect marriage negatively and divorce rates positively. In the face of an increased risk of getting infected, individuals exhibit emotions, such as aversion and anxiety, for self-protection (Schaller & Murray, 2008); this, in turn, can affect interpersonal relationships and create conflict in relationships. Conversely, *attachment theory* suggests that “stress leads to affiliation” and would have positive forces on marriage and relationships (Cohan & Cole, 2002, pg. 17).

Finally, natural disasters may be a close analog to the COVID-19 pandemic. Ahmed (2017), Hamamatsu, Inoue, Watanabe and Umezaki (2014), Prati and Pietrantoni (2014) found that marriage rates decreased in the year following earthquakes and floods. Cohan et al. (2009) showed divorce rates decreased following a major manmade disaster characterized by death. By contrast, Cicatiello et al. (2019), Cohan and Cole (2002), Xu and Feng (2016) found hurricanes and earthquakes to be associated with increases in both marriage and divorce rates. With regard to the timing of marriage, both male and female age at marriage was found to decrease as an aftereffect of earthquakes and floods in India and Nepal (Das & Dasgupta, 2020, Ebitt, 2015, Khanna & Kochhar, 2020).^12^ Therefore, there is mixed evidence on the impacts of natural disasters on the marriage and divorce rate depending on the scale, timing, and location of disasters. Due to the mixed findings, no clear indication of the potential impacts of the pandemic can be drawn from this literature.

## Background

### The COVID-19 pandemic in Mexico

**Fig. 1:**
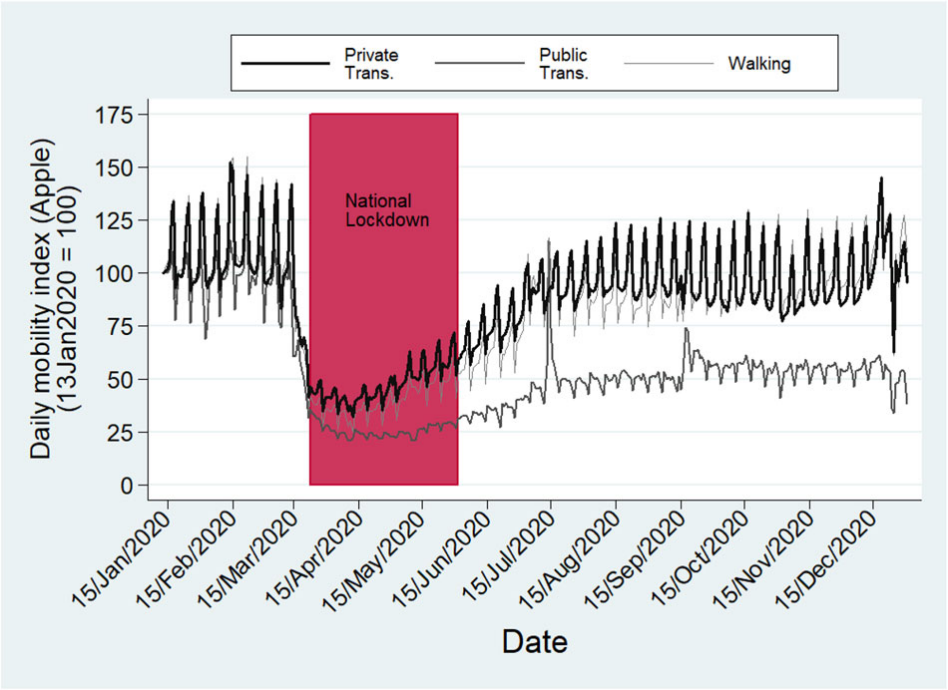
Background: mobility and national lockdown. SOURCE: Apple mobility reports. NOTES: The red area indicates the national stay-at-home order

The COVID-19 pandemic began in Mexico in March 2020. In March, Mexico’s federal government issued a national stay-at-home order, which closed schools and reduced mobility throughout the country.^13^ After two months of the national lockdown, the stay-at-home order was lifted on May 30th. At that point, though individual states were able to impose mobility restrictions, most economic activity restarted.^14^ Further, Fig. 1 shows the mobility patterns nationwide. Even though Mexico is a country where the government has inadequate power to enforce the stay-at-home order, Mexico’s mobility fell substantially for the duration of the national lockdown, suggesting that citizens complied with the regulations imposed by Mexico’s Federal Government. Once restrictions were left to each state, mobility patterns recovered slowly, although mobility never reached pre-pandemic levels during 2020.

During the lockdown, some state registrars and state courts closed.^15^ In the case of state registrars, the only states that closed for marriages and administrative divorces were Tabasco (April–June), Tlaxcala (May), Veracruz (April–May), and Yucatan (April–May). Even though the registrars were open for the remainder of the states, the celebration of marriages needed an appointment, with documents signed off-premises.

Conversely, state courts were closed in many states.^16^ States with court closures postponed marriages and divorces requiring a court resolution (Martinez, 2020, Radio, 2020, Trials, 2020). For the case of marriages requiring a court resolution, if a registrar was closed, but the court was open in a given state, the latter could mandate the former to proceed with the marriage registration, such as in the case of Veracruz. Figure A.2 presents the states that closed their registrars for marriages and administrative divorces (Panel A) and states that closed their civil courts (Panel B). These maps show that most state registrars and state courts remained open during the lockdown, although at a lower capacity (by appointment).

Further, relevant for contextualizing the household impact of the pandemic is the financial circumstances in Mexico during the pandemic. Mexican households were severely impacted in terms of income and unemployment over the course of 2020 (Hoehn-Velasco et al., 2021b, 2022). Despite these income and employment losses, the Mexican government failed to offer a safety net during the recession in response to this income loss (unlike high-income settings (Hale, Webster, Petherick, Phillips & Kira, 2020)). Employment in Mexico did recover over the course of 2020 (mostly in the informal sector). However, at the close of 2020, employment levels were still below previous years (Hoehn-Velasco et al., 2021b, 2022).

### Marriage and divorce in Mexico

Marriage rates in Mexico have been on a secular decline for several decades (Hoehn-Velasco & Penglase, 2021a). This trend has been coupled with rising non-marital births (Hoehn-Velasco & Penglase, 2021a), suggesting that many couples may be choosing not to formalize their union through marriage. Further, educational attainment has increased substantially in Mexico (Hoehn-Velasco & Penglase, 2021a). This higher educational attainment likely contributed to the national rise in the average age at first marriage (for both members of the couple). Another important factor in this rise in average marriage age was the amendment of the Federal Civil Code which changed the legal age of marriage to 18 years of age, without exceptions or legal exemptions at the federal and local levels (de Gobernación, 2019).^17^

At the same time as marriage has declined, divorce rates have been increasing (García-Ramos, 2021, Hoehn-Velasco & Penglase, 2021c). A major causal contributor to the rise in divorce rates has been the legal adoption of no-fault unilateral divorce in Mexico.^18^ However, other factors may be at play, including increasing educational attainment, higher female labor force participation, and changing social norms toward divorce (Hoehn-Velasco & Penglase, 2021a, c).

**Fig. 2:**
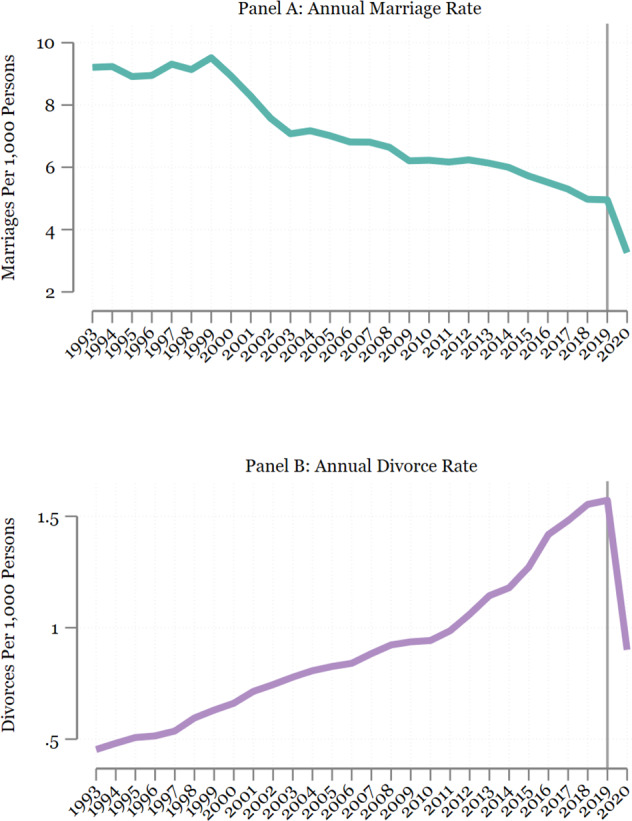
Background: marriage and divorce rates over time. Source: INEGI marriage and divorce microdata. Notes: The vertical dashed line indicates the COVID-19 pandemic

Figure 2 Panels A and B show national secular trends in marriage and divorce rates from 1993 to 2020.^19^ In Panel A, over the two-and-a-half decades presented, marriage rates decline by 45%. Divorce rates more than double over the same period. The clearest rise in the divorce rate occurs after unilateral no-fault divorce began to spread throughout Mexico. Unilateral divorce began in Mexico City in 2008 and then gradually spread to all other Mexican states by 2017 (Hoehn-Velasco & Penglase, 2021c).

## INEGI divorce and marriage records

To study the marriage and divorce rates, we use divorce and marriage microdata available from the Instituto Nacional de Estadística y Geografía (INEGI). For the analysis, we include the years 2017–2020, with 2020 as the year of focus. Earlier years, 2017–2019, are included as control years to gauge the counterfactual if the pandemic had not occurred.^20^ A limitation of this data, is that the latest available data only includes the first wave of the COVID-19 pandemic (through 2020); hence, our estimates provide the initial short-run effect over 2020.

The INEGI data over this timespan includes all individual records for each divorce and marriage that occurred in Mexico. The data includes detailed characteristics, including where and when the divorce (and marriage) occurred and the type of divorce (and marriage). The data also report individual characteristics of the husband and wife, including employment, education, and age. We aggregate this data to the state level, and our final sample includes 1536 observations (32 states x 12 months x 4 years).

For the type of divorce, divorces can occur via a judicial or administrative process. Administrative divorces are divorces where the couple jointly files and can only occur in marriages that do not have minor children or substantial asset division required. In addition to administrative versus judicial divorces, divorces are also categorized by cause. Divorces can be unilateral, with mutual consent, or with cause (Hoehn-Velasco & Penglase, 2021c). At this point (post-unilateral divorce), divorces occurring in Mexico are generally mutual consent or unilateral divorces rather than divorces stating a specific cause. However, INEGI does include 28 causes of divorce (including mutual consent and unilateral divorce).^21^ In addition to the characteristics of the divorce, the divorce records also include information on the marriage, including when and where the marriage occurred.

One complicating issue with the divorce records is the date of the divorce. For our analysis, we use the date the divorce was executed because this date aligns with the year of the record (e.g., 2020). However, the INEGI data records three dates for each divorce. The *execution* date, the divorce *registration* date, and the *sentencing* date. While the pandemic may have also affected filings and sentencing, we view the divorce executions as the best representative date of when divorce was finalized.^22^

The marriage records contain similar information to divorce records. In the marriage records, the marriage type refers to the chosen property division. Property division types include communal, separate, or mixed property. Most marriages fall under shared/communal property (two-thirds of marriages, Hoehn-Velasco and Penglase (2021c)). Marriages with communal property share assets across spouses, while separate property divides assets across the spouses. Further, the marriage records also include the date the marriage occurred and the characteristics of spouses entering the marriage.

### Summary statistics

**Fig. 3:**
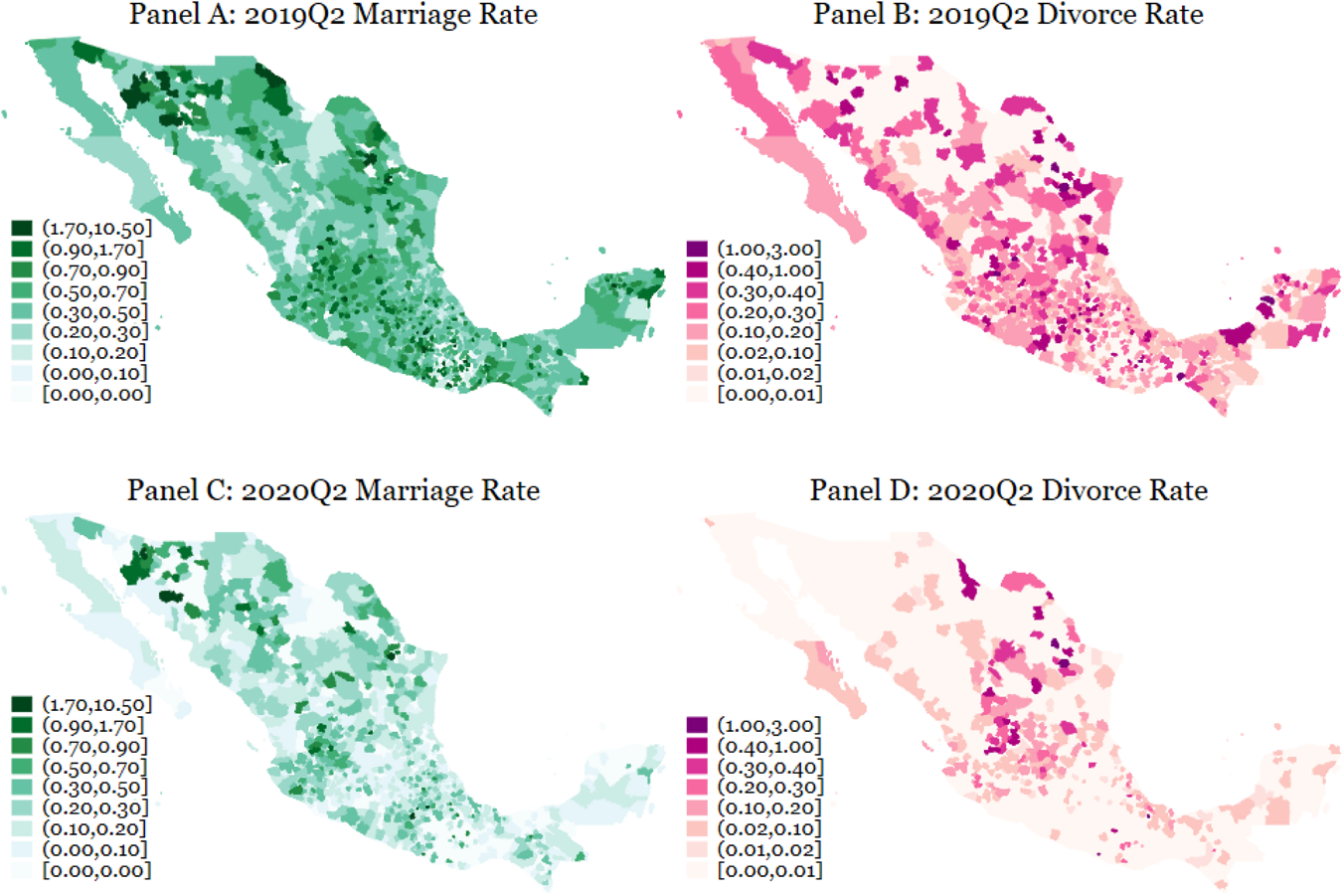
Map of change in divorce and marriage rates, 2020Q2 v. 2019Q2. Source: INEGI marriage and divorce microdata

**Fig. 4:**
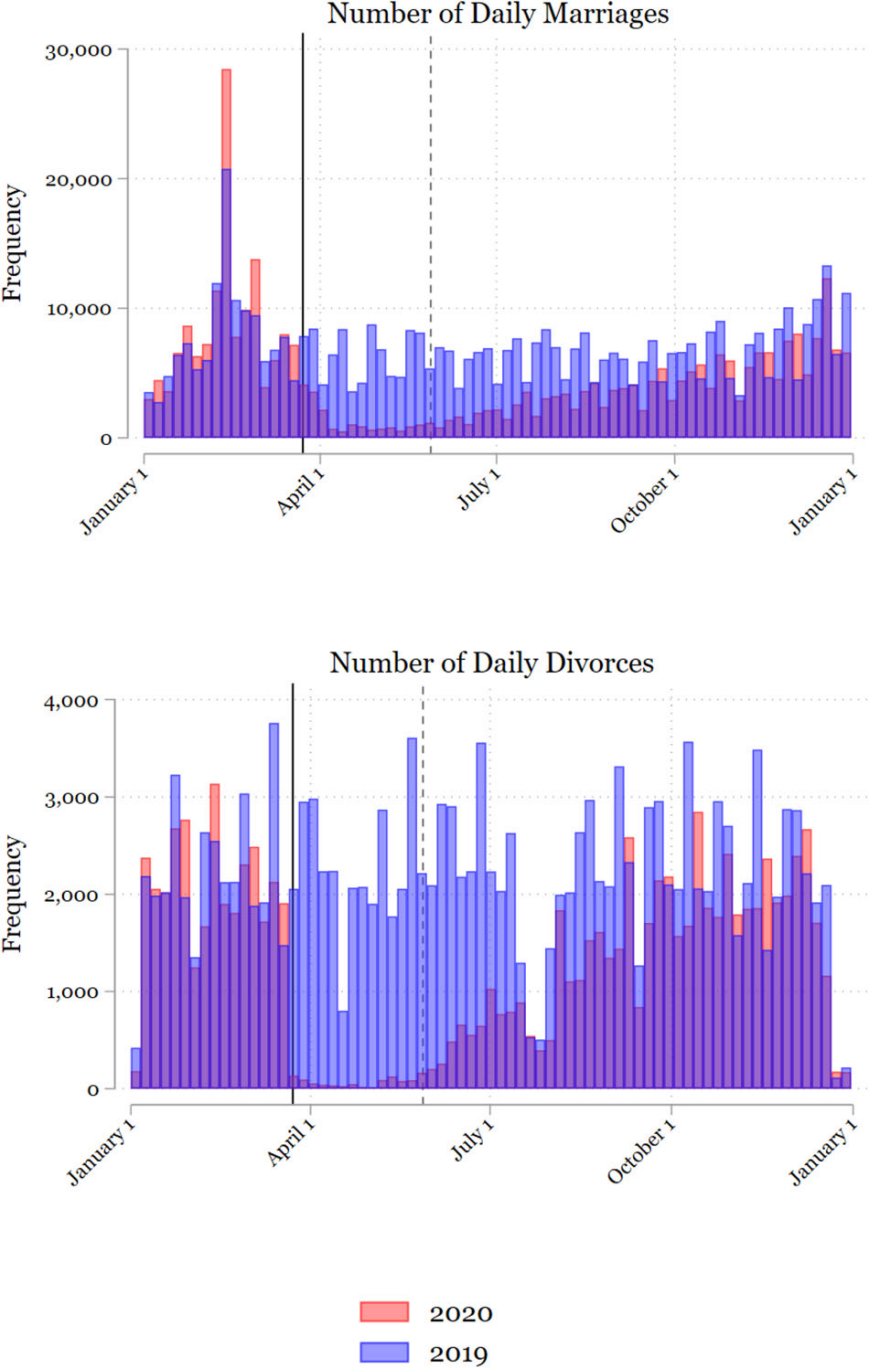
Volume of marriages and divorces, 2020 v. 2019. Source: INEGI marriage and divorce microdata. Notes: The vertical lines indicate the start date (solid black) and the end date (dashed black) of the stay-at-home order. The spike in marriages in 2020 occurs on February 14th (Valentine’s Day). Red shows 2020, and blue shows 2019. Purple is the overlapping bars for 2020 and 2019 (the combination of the transparent blue and red bars)

First, we illustrate the spatial drop in marriage and divorce rates throughout Mexico. Figure 3 presents the municipal-level map of marriage and divorce rates over the second quarter of 2020 and 2019. Over 2020 there is a clear drop in marriage and divorce rates, apparent throughout Mexico. This drop in marriages is reflected in Figure 4, which displays the marriage and divorce counts by date. There is a clear drop-off in both the number of marriages and divorces during the lockdown period of 2020 (illustrated by the vertical lines) but divorces clearly recover afterward.

Second, Table 1 displays the summary statistics over the pre and post-pandemic period. The pre-pandemic period includes 2017m1-2020m2. The post-pandemic period includes 2020m3-2020m12. Here, the marriage and divorce rates are presented per 1000 persons aged 15 and over.^23^ At the top of Table 1, after the pandemic begins, marriage rates declined by 23.6 per 1000, and divorce rates drop by 6.7 per 1000. Underneath these aggregate rates, we also show divorce and marriage rates by the wife’s age (per population of women in each age group). The largest declines in linear marriage rates occur for those 15–29, but in percentage terms, similar declines occur for all groups under 65. By age, divorces decline the most for wives between 30 and 44.^24^

**Table 1:** Summary statistics

	Pre-Post Pandemic				
	Pre-COVID		Post-COVID		Pre-Post
	Mean	Std. Dev.	Mean	Std. Dev.	Diff.
Overall					
Divorce Rate	0.153	0.083	0.086	0.088	−0.067***
Marriage Rate	0.491	0.217	0.255	0.190	−0.236***
Marriage Rate-Age					
Rate Wife 15–29	1.738	0.749	0.905	0.701	−0.832***
Rate Wife 30–44	0.863	0.426	0.459	0.355	−0.404***
Rate Wife 45–64	0.316	0.207	0.173	0.131	−0.143***
Rate Wife 65+	0.077	0.067	0.041	0.038	−0.037***
Rate Husband 15–29	1.472	0.649	0.757	0.590	−0.715***
Rate Husband 30–44	1.157	0.545	0.607	0.465	−0.550***
Rate Husband 45–64	0.463	0.284	0.252	0.188	−0.210***
Rate Husband 65+	0.265	0.175	0.150	0.113	−0.114***
Divorce Rate-Age					
Rate Wife 15–29	0.178	0.117	0.095	0.110	−0.082***
Rate Wife 30–44	0.448	0.256	0.252	0.265	−0.195***
Rate Wife 45–64	0.268	0.147	0.150	0.154	−0.118***
Rate Wife 65+	0.047	0.037	0.026	0.032	−0.021***
Rate Husband 15–29	0.121	0.083	0.064	0.076	−0.057***
Rate Husband 30–44	0.482	0.266	0.270	0.283	−0.212***
Rate Husband 45–64	0.359	0.189	0.200	0.202	−0.159***
Rate Husband 65+	0.105	0.069	0.059	0.065	−0.046***
% Marriages-Educ					
Wife Primary	11.291	5.639	8.753	6.042	−2.538***
Wife Middle	27.938	6.267	23.551	8.627	−4.387***
Wife Secondary	28.892	4.455	29.479	6.594	0.587
Wife Higher-Ed	30.588	8.167	36.787	11.148	6.199***
Husband Primary	13.063	5.588	10.310	5.828	−2.753***
Husband Middle	28.088	6.043	23.857	7.613	−4.231***
Husband Secondary	28.200	5.006	29.613	6.669	1.413***
Husband Higher-Ed	29.456	7.765	34.826	11.829	5.370***
% Divorce-Educ					
Wife Primary	13.164	7.310	12.907	15.665	−0.258
Wife Middle	30.367	7.962	28.734	15.625	−1.632*
Wife Secondary	25.699	6.961	26.196	15.426	0.497
Wife Higher-Ed	29.952	8.887	31.066	17.078	1.114
Husband Primary	14.050	7.300	15.065	17.632	1.015
Husband Middle	29.468	7.351	27.736	15.292	−1.732*
Husband Secondary	27.039	7.109	26.392	14.847	−0.647
Husband Higher-Ed	28.640	8.395	29.622	17.606	0.982
% Marriages-Type					
Shared Property Division	60.673	30.932	63.063	27.960	2.390
Separate Property Division	32.353	27.846	35.011	27.657	2.658
Mixed Property Division	0.003	0.031	0.002	0.017	−0.001
% Divorce-Cause					
Unilateral	54.701	30.304	51.782	35.386	−2.919
Mutual Consent	38.894	25.994	46.238	34.810	7.344***
With Cause	6.116	8.881	1.747	6.722	−4.369***
% Divorce-Type					
Judicial	89.587	13.909	74.174	35.293	−15.413***
Admin	10.413	13.909	25.826	35.293	15.413***
*N*	1216		320		1536

Source: INEGI marriage and divorce microdata

Notes: The divorce and marriage rates are reported per 1000 persons 15 and over. Age-and-sex-specific rates are per 1000 persons of that population. The percentage outcomes are per 100 marriages or divorces. Education at the primary level is defined as a primary education or less. Higher education is defined as college or technical education

***, * represent statistical significance at 1 and 10 percent levels

Next, we show the percentage of divorces and marriages. Marriages to women with higher levels of education increase after the pandemic, while unions for those with either a primary education (or less) or a middle-school education decline.^25^ In the case of divorce, divorces for women with middle school education decrease by 1.6 percentage points. The percentage of marriages with shared property division increase from 60.7% to 63%.^26^,^27^

## Empirical strategy

We consider the impact of the COVID-19 pandemic on marriage and divorce rates using both an event-study design and a difference-in-differences specification. In our analysis, we lack a never-treated “control group” in each specification, as the pandemic hit all states in Mexico simultaneously. Thus, our main specification compares the impact of the 2020 COVID-19 pandemic (the treatment year), to control years 2017–2019. The event study compares the post-pandemic months (March–December) relative to pre-pandemic months in 2020, as well as to control years.

In other words, since the onset of the pandemic was in March 2020, we exploit the intertemporal variation in marriage rates and divorce rates over the pre and post-period of the pandemic year (i.e., 2020) relative to the same months in the non-pandemic years (i.e., 2017–2019). This comparison provides two levels of variation. First, over the months leading up to the onset of the pandemic, i.e., January–February to those in the months after, i.e., March–December in 2020. The second level of variation comes from comparing the year the COVID-19 pandemic hit the world, i.e., 2020, to the pre-pandemic years 2017–2019. This empirical strategy follows related published work, including Bullinger et al. (2020), Leslie and Wilson (2020), Hoehn-Velasco et al. (2021a), and de la Miyar et al. (2021). We also present a visual depiction of this methodology (for clarity) in A.1.

### Event-study specification

First, we use an event study to show the dynamic effect of the COVID-19 pandemic on marriage and divorce rates. The event study shows the month-by-month effect of the pandemic, which yields two advantages over the traditional difference-in-differences specification. First, plotting the pre-pandemic months allows us to directly test the parallel trends assumption using the months leading up to the pandemic.^28^ Second, the event study allows us to observe the month-by-month impact of the pandemic, which is essential to consider in our setting. During the pandemic, many outcomes likely follow a U-shape recovery (de la Miyar et al., 2021, Hoehn-Velasco et al., 2021a, Silverio-Murillo, de la Miyar & Hoehn-Velasco, 2020, 2021), and marriage and divorce rates likely follow a similar pattern. With a U-shaped recovery, the estimated impact of the pandemic on divorce and marriage rates will be downward biased and potentially close to zero (Goodman-Bacon & Marcus, 2020, Wolfers, 2006).

Formally, the event-study approach appears as:

1 $${\mathrm{y}}_{smy}=\alpha + \sum\limits_{ {q\neq-1}\atop{q=-8}}^{9} \beta_{q} {\mathrm{COVID}}-19_{yq}+\eta_{y} +\gamma_{m}+a_{s}+e_{smy}$$

where y_*smy*_ is the outcome of interest for state *s* in month *m* and year *y*. Our main outcomes of interest include the marriage rate (per 1000 persons 15 and over) as well as the divorce rate (per 1000 persons 15 and over). In the main results, we focus on linear rates, but we show the log of the marriage and divorce rates in the appendix.

COVID-19_*yq*_ represents a set of event-study style dummy variables. These dummy variables take a value of one in each respective period before and after the pandemic and are zero otherwise. In the above specification, March 2020 represents the first month of the post-pandemic period (*q* = 0). The final post-period represents December 2020, *q* = 9. We omit the month before the start of the pandemic, February 2020, or *q* = − 1 to avoid multicollinearity. Because the pandemic and stay-at-home order affected all states in Mexico simultaneously, we do not have staggered treatment timing. Instead, all months in the event study are compared against prior (control) years.

For the remainder of the specification, *η*_*y*_ are year fixed effects (our treatment groups). We include *γ*_*m*_ to compare the impact of the pandemic over each month, and account for seasonality. *a*_*s*_ are state-fixed effects. *e*_*smy*_ represents the regression error. We cluster standard errors at the state level. Because monthly time-varying controls are limited during the pandemic, and time-varying controls are known to cause issues in event-studies (Powell, 2021), we omit controls from our main specification. However, we consider pre-treatment levels of controls interacted with trends as a robustness check.

### Difference-in-differences specification

Second, we use a difference-in-differences design to compare the impact of the total effect of the pandemic relative to prior years. The difference-in-differences specification gives the average effect over the entire post-period, which in our case, yields the average impact of the pandemic on family formation and dissolution.

More specifically, our difference-in-differences specification compares the post-pandemic period in 2020 to the same months over earlier years, 2017–2019. Formally, this appears as:

2 $${{{{\rm{y}}}}}_{smy}=\alpha +\beta \,{1({{{\rm{COVID}}}}-19)}_{my}+{\eta }_{y}+{\gamma }_{m}+{a}_{s}+{e}_{smy}$$

where, again, y_*smy*_ represents the outcome, which includes marriage and divorce rates. COVID–19_*my*_ represents a binary variable capturing the post-pandemic period, which equals one for March 2020 to December 2020. As in the above equation, *η*_*y*_ are year fixed effects, *γ*_*m*_ are month-specific fixed effects, and *a*_*s*_ are state-fixed effects. *e*_*smy*_ represents the standard error, which we cluster at the state level, but also provide the Wild Cluster Bootstrapped p-values in the main tables (Cameron, Gelbach & Miller, 2008).

## Results

### Event-study findings

**Fig. 5:**
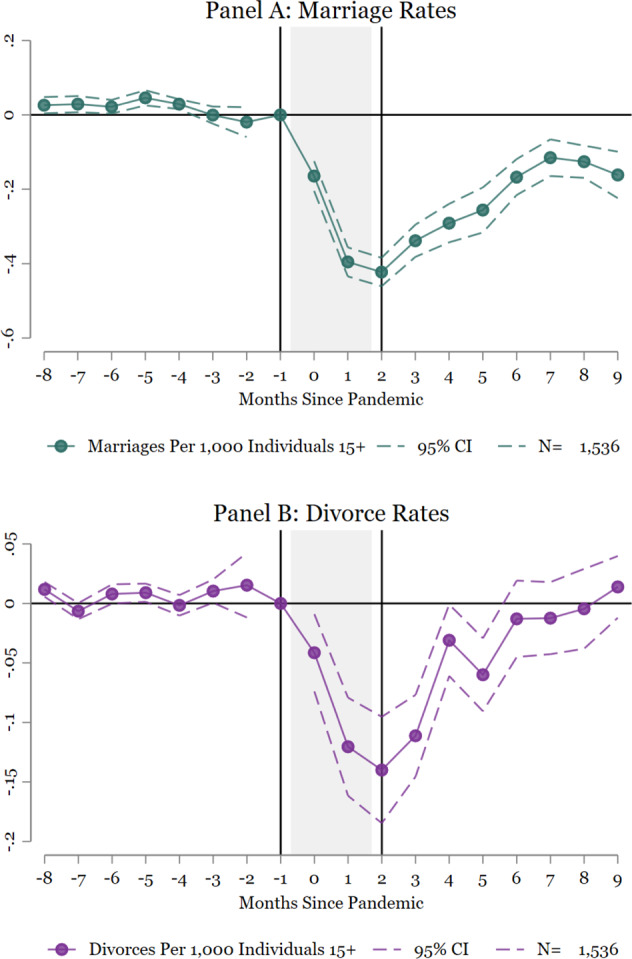
Event study results: marriage and divorce rates. Source: INEGI marriage and divorce microdata. Notes: Plotted coefficients are event-study dummy variables, *β*_*q*_. Each plotted point represents the number of months before and after the start of the pandemic. The event study considers 2017–2020, with 2020m2 as the omitted period. Solid lines represent point estimates. Dotted lines display the 95 percent confidence intervals. Baseline fixed effects include the state, month, and year. The divorce and marriage rates are reported per 1000 persons 15 and over. Results weighted by the state population 15 and over. Robust standard errors are clustered at the state level

Figure 5 presents the event-study results from Equation (1). The marriage rate is shown in Panel A (green), and the divorce rate in Panel B (purple). After the start of the pandemic (and stay-at-home order), both the state-level divorce rates and marriage rates decline over Panels A and B. Marriage rates decline by almost 90% in April and May of 2020 based on the monthly pre-pandemic mean (0.47). Divorces decline by 98% at the trough (May 2020), based on the pre-pandemic mean (0.14).

Divorce rates and marriage rates begin to recover after the stay-at-home order ends (indicated by the gray line). Divorce rates have fully recovered by September 2020 and are at baseline levels (based on prior years). Still, the divorce rates only recover and do not appear to boom, indicating that the pandemic still produced a net reduction in total divorces. In Panel A, marriage rates recover, but less so than divorce rates. Marriage rates are still 34% below original levels at the end of 2020.

We also show the results in logs in Figure A.5. The percentage declines between marriage rates and divorce rates are fairly similar. However, the divorce rate initially declines by more over the lockdown period. After the lockdown, state-level divorce rates recover faster than marriage rates, a finding that is similar to the linear specification. By September 2020, divorce rates have fully recovered, while marriage rates are still depressed below baseline levels at the end of 2020. Likely some of these marriages are being postponed until the COVID-19 pandemic entirely ends. However, we cannot determine whether the marriage rate will stay permanently depressed. While certain marriages may be postponed until lower COVID-19 case rates, other marriages may never occur. Further, pre-marriage coupling may be reduced due to lower social contact during the pandemic.

One limitation of this analysis is that we cannot observe the cause of the immediate dip in marriage and divorce rates. This drop may be attributable to a couple-specific choice to postpone the marriage/divorce. Or instead, it may be due to the closure of courts and limited ability to gather during the pandemic (Goldberg et al., 2021, Wagner et al., 2020). These two possible explanations have different interpretations and implications for future demographic trends. While we briefly try to disentangle these two explanations in Section 6.3, we are unable to fully determine the underlying mechanisms of the drop in marriages and divorces.

### Difference-in-differences results

Then, we turn to the difference-in-difference results in Table 2. The coefficients in this table represent the average monthly decline in divorce and marriage rates over 2020 relative to prior years. In addition to the linear rates, we present the mean over 2019 from the same months, March through December, and the percentage change based on that mean.

**Table 2:** Difference-in-differences results: divorce and marriage rates

RATE PER 1000:	Marriages				Divorces			
	(1)	(2)	(3)	(4)	(5)	(6)	(7)	(8)
	Marriage Rate	Separate Property Rate	Shared Property Rate	Divorce Rate	Admin Divorce Rate	Judicial Divorce Rate	Mutual Divorce Rate	Unilateral Divorce Rate
1(COVID-19)	−0.24***	−0.08***	−0.16***	−0.06***	−0.01**	−0.06***	−0.02***	−0.04***
	(0.01)	(0.02)	(0.01)	(0.01)	(0.00)	(0.01)	(0.00)	(0.01)
*N*	1536	1536	1536	1536	1536	1536	1536	1536
Adjusted R-squared	0.56	0.79	0.61	0.79	0.80	0.81	0.82	0.82
Wild Bootstrap *P*-Value	0.00	0.00	0.00	0.00	0.00	0.00	0.00	0.00
2019 March–December Mean	0.44	0.15	0.27	0.14	0.01	0.13	0.05	0.09
COVID-19 Percentage Change	−54.3%	−55.4%	−57.9%	−43.4%	−52.6%	−42.6%	−39.4%	−45.7%
Baseline FE	X	X	X	X	X	X	X	X

Source: INEGI marriage and divorce microdata

Notes: Post-COVID is a dummy variable capturing the impact of the pandemic, which equals one from March 2020 to December 2020. Baseline fixed effects include the state, month, and year. The divorce and marriage rates are reported per 1000 persons 15 and over. Results weighted by the state population 15 and over. Robust standard errors are clustered at the state level

***, ** represent statistical significance at 1 and 5 percent levels

In Column (1), marriages declined by 0.24 marriages per 1000 persons, representing a 54.3% decline from the 2019 mean. In addition to the overall effect on marriage rates, we split the marriage rate into couples choosing separate and combined property. Both types of marriages decline by a similar amount, with shared property declining slightly more in percentage terms (over Columns (2) and (3)).

For divorce rates, in Column (4), the divorce rate falls by 0.06 or 43.4% of the 2019 mean. Similar to marriages, we also split the divorce rates by type over Columns (5)–(8). For the method of divorce, judicial versus administrative divorce, administrative divorces decline by less in the overall point estimate, but by more in percentage terms (52.6% versus 42.6%). While this may seem counter-intuitive, when considering these results in an event study in Appendix Figure A.6, judicial divorces show a sharp reduction during the initial stay-at-home order, then judicial divorces quickly recover. Mutual consent divorces (requiring both members of the couple to agree to the divorce) decline by the least, 39.4%, and unilateral divorces decline by more, 45.7%.

### Robustness

We also show several robustness checks on the difference-in-differences results in Table A.3. First, we add several controls based on 2015 census levels interacted with linear trends. The additional controls include the educational levels and sex ratios. These controls have little impact on the point estimates. Second, we add month-by-year linear trends to the results. With these monthly trends, the estimates remain similar to the baseline. Third, we remove Mexico City from the results, which does reduce the estimate slightly, but the findings are similar to the baseline. Fourth, in our main results, we include states with no marriages or divorces as zeros. But here, we replace these zero records with missing observations to ensure the zeros are not driving the results. When we replace zeros as missing, the results are the same as the baseline. This also suggests that the results are not entirely driven by court closures. Fifth, we aggregate to the national level and consider the effect of the pandemic without state-level effects. At the national level, the results are the same as the baseline.

Finally, for divorces in Table A.3, we use the date registered rather than the date executed. Using the date of divorce registration shows a more modest decline during the pandemic. Registered divorces only drop by 28%, versus the 43% baseline. While individuals may not have been able to obtain a divorce, they continued registering during the pandemic. Overall, beyond the alternative definition of divorce registrations, the results are insensitive to alternative specifications. Our findings indicate a large decline in both marriage and divorce rates that is not dependent on the inclusion of controls, trends, or other adjustments to the specification.

## Mechanisms: heterogeneous effects and compositional changes

### Couple characteristics: age

**Fig. 6:**
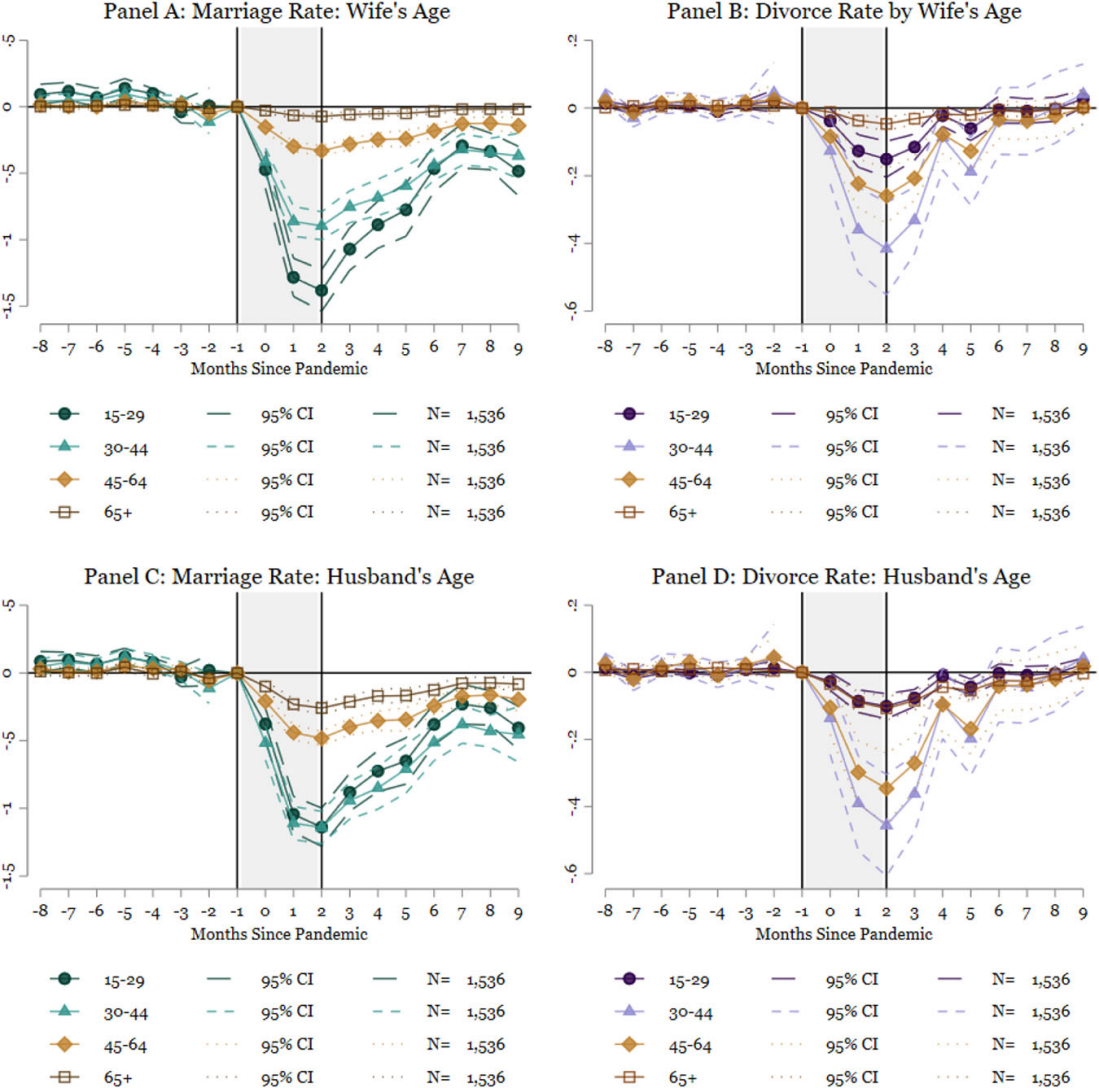
Marriage and divorce rates by husband and wife’s age. Source: INEGI marriage and divorce microdata. Notes: Plotted coefficients are event-study dummy variables, *β*_*q*_. Each plotted point represents the number of months before and after the start of the pandemic. The event study considers 2017–2020, with 2020m2 as the omitted period. Solid lines represent point estimates. Dotted lines display the 95 percent confidence intervals. Baseline fixed effects include the state, month, and year. Each rate of marriage and divorce by age-sex group is per 1000 persons in that age-sex population. Weights applied by the age-sex population. Robust standard errors are clustered at the state level

Figure 6 shows the impact of the pandemic on age-specific marriage and divorce rates. We calculate the population in each age group for men and women separately and then calculate the marriage and divorce rates per 1000 persons in each age-sex group. In Panel A, the most considerable reduction in the marriage rate occurs for women ages 15 to 29. Marriage rates for women between 30 and 44 also show a decrease but to a lesser extent. For men, as seen in Panel C, the reduction in the marriage rate is symmetric for those between 15 to 19 and 30 to 44. Men over 45 show a modest decline in marriage rates, while women over 65 show no change in marriage rates, reflecting the lower marriage rates for women in this age group.

For divorce rates in Panels B and D, the largest reduction in divorce rates occurs for men and women between 30 and 44. Individuals over 45 experience the next largest decline in divorce rates. Those ages 15 to 29 show less of a drop, likely because these individuals had relatively low divorce rates to begin with (based on Table 1).

To separate the compositional effects, we consider the percent of marriages and divorces by couple characteristics (rather than rates). Table 3 presents the difference-in-differences specification over the percent of marriages and divorces by the wife’s age and the husband’s age. The results represent the compositional change in new marriages and divorces that occurred during the pandemic.

**Table 3:** Percentage of marriage and divorces by age and education

Panel A: Percentage of Marriages by Age								
% OF MARRIAGES:	Wife				Husband			
	(1)	(2)	(3)	(4)	(5)	(6)	(7)	(8)
	15–29	30–44	45–64	65+	15–29	30–44	45–64	65+
1(COVID-19)	1.79***	−1.45***	−0.40**	0.06	1.52***	−1.33***	−0.40*	0.22**
	(0.37)	(0.28)	(0.18)	(0.05)	(0.32)	(0.26)	(0.21)	(0.08)
*N*	1532	1532	1532	1532	1532	1532	1532	1532
Adjusted R-squared	0.91	0.90	0.84	0.57	0.91	0.87	0.84	0.69
Wild Bootstrap *P*-Value	0.00	0.00	0.02	0.26	0.00	0.00	0.06	0.01
2019 March–December Mean	61.49	28.52	9.14	0.86	51.60	33.95	11.93	2.51
COVID-19 Percentage Change	2.9%	−5.1%	−4.4%	6.9%	2.9%	−3.9%	−3.4%	8.6%
Baseline FE	X	X	X	X	X	X	X	X

Panel B: Percentage of Divorces by Age								
% OF DIVORCES:	Wife				Husband			
	(1)	(2)	(3)	(4)	(5)	(6)	(7)	(8)
	15–29	30–44	45–64	65+	15–29	30–44	45–64	65+
1(COVID-19)	0.82**	0.68	−1.30***	−0.20	0.75***	0.63	−1.06**	−0.31
	(0.33)	(0.52)	(0.36)	(0.13)	(0.27)	(0.40)	(0.40)	(0.20)
*N*	1513	1513	1513	1513	1513	1513	1513	1513
Adjusted R-squared	0.70	0.35	0.62	0.30	0.70	0.35	0.59	0.38
Wild Bootstrap *P*-Value	0.02	0.21	0.00	0.14	0.01	0.17	0.03	0.15
2019 March–December Mean	21.30	49.13	27.70	1.88	14.58	48.39	33.43	3.59
COVID-19 Percentage Change	3.9%	1.4%	−4.7%	−10.7%	5.1%	1.3%	−3.2%	−8.7%
Baseline FE	X	X	X	X	X	X	X	X

Panel C: Percentage of Marriages by Education								
% OF MARRIAGES:	Wife				Husband			
	(1)	(2)	(3)	(4)	(5)	(6)	(7)	(8)
	Primary	Middle	Secon-dary	College (or Higher)	Primary	Middle	Secon-dary	College (or Higher)
1(COVID-19)	−0.42	−2.85***	0.31	2.99***	−0.47	−2.33***	0.23	2.59***
	(0.40)	(0.79)	(0.61)	(0.86)	(0.32)	(0.75)	(0.42)	(0.86)
*N*	1532	1532	1532	1532	1532	1532	1532	1532
Adjusted R-squared	0.80	0.68	0.61	0.75	0.80	0.70	0.74	0.74
Wild Bootstrap *P*-Value	0.33	0.00	0.62	0.00	0.15	0.01	0.61	0.01
2019 March–December Mean	10.48	27.68	29.48	31.09	12.07	27.57	29.36	29.80
COVID-19 Percentage Change	−4.0%	−10.3%	1.0%	9.6%	−3.9%	−8.5%	0.8%	8.7%
Baseline FE	X	X	X	X	X	X	X	X

Panel D: Percentage of Divorces by Education								
% OF DIVORCES:	Wife				Husband			
	(1)	(2)	(3)	(4)	(5)	(6)	(7)	(8)
	Primary	Middle	Secon-dary	College (or Higher)	Primary	Middle	Secon-dary	College (or Higher)
1(COVID-19)	1.62*	–3.49***	0.76	0.36	1.33	–2.84***	0.44	0.38
	(0.94)	(0.96)	(1.52)	(0.78)	(1.07)	(0.86)	(1.81)	(1.09)
*N*	1499	1499	1499	1499	1498	1498	1498	1498
Adjusted R-squared	0.72	0.68	0.42	0.73	0.70	0.63	0.40	0.71
Wild Bootstrap *P*-Value	0.14	0.00	0.68	0.64	0.29	0.01	0.82	0.75
2019 March–December Mean	11.58	31.10	26.01	30.81	12.57	29.90	27.61	29.41
COVID-19 Percentage Change	14.0%	−11.2%	2.9%	1.2%	10.6%	–9.5%	1.6%	1.3%
Baseline FE	X	X	X	X	X	X	X	X

Source: INEGI marriage and divorce microdata

Notes: Post-COVID is a dummy variable capturing the impact of the pandemic, which equals one from March 2020 to December 2020. Baseline fixed effects include the state, month, and year. The percentage outcomes are per 100 marriages or divorces. Results weighted by the number of marriages/divorces. Robust standard errors are clustered at the state level. Significance levels reported at the 10, 5, and 1 percent levels. Education at the primary level is defined as a primary education or less. Higher education is defined as college or technical education

Across both Panels A and B, the largest relative increase occurs for those between 15 and 29 years of age. Younger individuals appear more willing to continue plans to marry and divorce, despite the ongoing pandemic. By contrast, the largest percentage reduction in marriages (for both men and women appears) for those between 30 and 64. Panel B shows that the clearest percentage reduction in divorces appears for those between 45 and 64.

### Couple characteristics: education

**Fig. 7:**
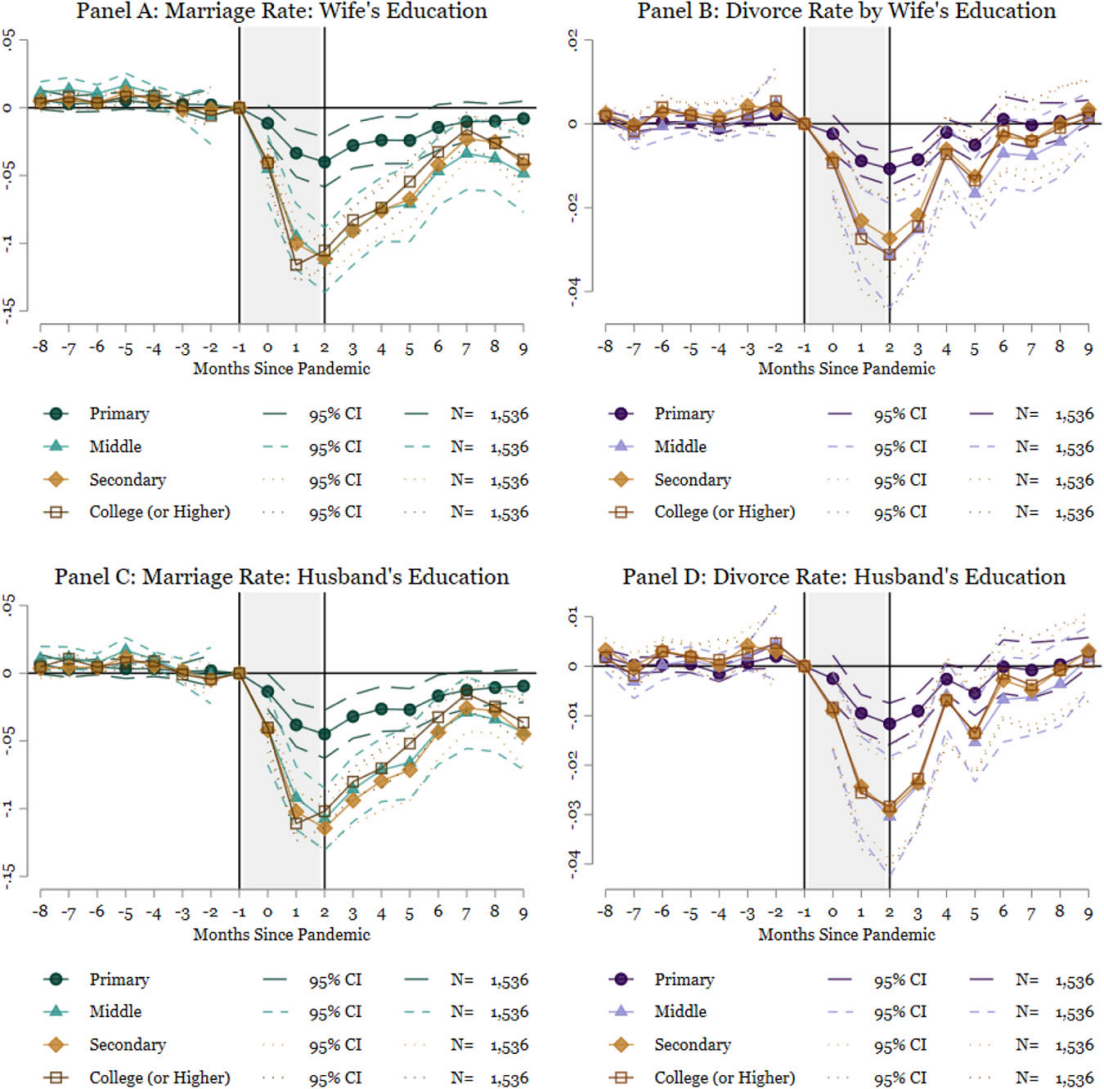
Marriage and divorce rates by husband and wife’s education. Source: INEGI marriage and divorce microdata. Notes: Plotted coefficients are event-study dummy variables, *β*_*q*_. Each plotted point represents the number of months before and after the start of the pandemic. The event study considers 2017–2020, with 2020m2 as the omitted period. Solid lines represent point estimates. Dotted lines display the 95 percent confidence intervals. Baseline fixed effects include the state, month, and year. The percentage outcomes are per 100 marriages or divorces. Results weighted by the number of marriages/divorces. Robust standard errors are clustered at the state level

Next, in Fig. 7, we show the change in marriage and divorce rates by education. Here we use the total population, 15 and over, as the denominator. In Panels A and C, marriage rates decline for all levels of education, except for those with a primary education or less. The results appear similar for divorce rates in Panels B and D.

Then, we consider the compositional effects (percentage terms) in Panels C and D of Table 3. Panel C shows that those with higher levels of education (college or technical education) display a relative increase in new marriages during the pandemic. Divorces show the opposite pattern, where divorces to those with a primary education (and less) show the largest gains in percentage terms (mainly for the wife). To further examine this change to educational composition, in the Appendix (see Table A.4), we also consider whether couples are more likely to marry or divorce within their shared educational category during the pandemic. We consider a binary variable capturing whether each member of the couple has the same level of educational attainment for any shared education level, shared primary, shared middle, shared secondary, and shared higher education. The results suggest that new marriages shift towards those with a college education (or higher), and new divorces shift towards those with a primary education. These findings reflect the compositional changes of new marriages and divorces from Table 3. However, a limitation of these findings is that they are only suggestive, and we do not adopt a formal method of measuring assortativeness such as in Chiappori, Dias and Meghir (2020).

### Couple characteristics: employment and other factors

Then, we consider other related couple characteristics that may have adjusted during the pandemic. First, in Table 4 Panel A, we consider the heterogeneous impact by the employment status of the husband and wife. For new marriages, there is little change in the employment status of the husband and wife. This failure of marriages to shift towards employed or unemployed spouses is surprising. Since the employment losses were significant in Mexico (Hoehn-Velasco et al., 2022), we might expect marriages to employed spouses to decline. By contrast, unemployed spouses may be more likely to delay marriage because of economic uncertainty and income loss. The data suggest that neither of these two possibilities is plausible, or potentially, any impact canceled itself out on average. While marriages do not shift in employment composition, divorces do shift. Divorces where the wife is employed and where both spouses are employed increase during the pandemic. Thus, divorces shifted towards employed wives but not husbands; this change can be linked to the increased ability of economically self-sufficient women to leave dissatisfying, undesirable marital unions (Lehrer & Son, 2017).

**Table 4:** Percentage of marriage and divorces by other characteristics

Panel A: Employment						
% WHERE WIFE/HUSBAND EMPLOYED	Marriages			Divorces		
	(1)	(2)	(3)	(4)	(5)	(6)
	Wife	Husband	Both	Wife	Husband	Both
1(COVID-19)	0.78	−0.05	−0.01	2.95*	−0.21*	2.83**
	(1.52)	(0.48)	(1.91)	(1.68)	(0.11)	(1.32)
*N*	1499	1499	1513	1532	1532	1532
Adjusted R-squared	0.71	0.49	0.89	0.80	0.76	0.82
Wild Bootstrap *P*-Value	0.62	0.92	1.00	0.10	0.08	0.05
2019 March–December Mean	67.00	94.18	46.88	54.12	96.55	49.87
COVID-19 Percentage Change	1.2%	−0.1%	−0.0%	5.5%	−0.2%	5.7%
Baseline FE	X	X	X	X	X	X

Panel B: Same Sex, Children, and Who Initiates						
%	Marriages			Divorces		
	(1)	(2)	(3)	(4)	(5)	(6)
	Same Sex	Same Sex	Has Child	Husband Initiates	Wife Initiates	Both Initiate
1(COVID-19)	0.06	0.04	0.80	−0.95	−3.40***	4.35***
	(0.06)	(0.02)	(0.79)	(0.77)	(0.66)	(1.16)
*N*	1532	1513	1513	1513	1513	1513
Adjusted R-squared	0.90	0.78	0.81	0.87	0.92	0.93
Wild Bootstrap *P*-Value	0.38	0.16	0.32	0.20	0.00	0.00
2019 March–December Mean	0.77	0.22	49.08	25.89	33.71	40.40
COVID-19 Percentage Change	7.6%	16.4%	1.6%	−3.7%	−10.1%	10.8%
Baseline FE	X	X	X	X	X	X

Panel C: Type of Marriage and Divorce Characteristics						
%	Marriages			Divorces		
	(1)	(2)	(3)	(4)	(5)	(6)
	Separate	Shared	Admin.	Judicial	Mutual	Unilateral
1(COVID-19)	0.44	−0.14	1.66*	−1.66*	1.37	−2.86
	(0.62)	(0.67)	(0.88)	(0.88)	(2.18)	(2.38)
*N*	1532	1532	1513	1513	1513	1513
Adjusted R-squared	0.98	0.96	0.89	0.89	0.94	0.92
Wild Bootstrap *P*-Value	0.48	0.82	0.04	0.04	0.58	0.27
2019 March–December Mean	33.14	60.72	9.07	90.93	34.20	63.58
COVID-19 Percentage Change	1.3%	−0.2%	18.3%	−1.8%	4.0%	−4.5%
Baseline FE	X	X	X	X	X	X

Panel D: Children and Length of Marriage						
%	Divorces					
	(1)	(2)	(3)	(4)	(5)	(6)
	Has Child	Marriage Length <5	Marriage Length 5–10	Marriage Length 10–20	Marriage Length 20–30	Marriage Length >30
1(COVID-19)	0.80	1.40***	1.17***	−0.58	−1.20***	−0.81**
	(0.79)	(0.49)	(0.30)	(0.35)	(0.32)	(0.30)
*N*	1513	1513	1513	1513	1513	1513
Adjusted R-squared	0.81	0.57	0.16	0.50	0.33	0.47
Wild Bootstrap *P*-Value	0.32	0.01	0.00	0.12	0.00	0.01
2019 March–December Mean	49.08	16.26	20.16	29.09	21.60	12.39
COVID-19 Percentage Change	1.6%	8.6%	5.8%	−2.0%	−5.6%	−6.5%
Baseline FE	X	X	X	X	X	X

Source: INEGI marriage and divorce microdata

Notes: Post-COVID is a dummy variable capturing the impact of the pandemic, which equals one from March 2020 to December 2020. Baseline fixed effects include the state, month, and year. The percentage outcomes are per 100 marriages or divorces. Results weighted by the number of marriages/divorces. Robust standard errors are clustered at the state level. Significance levels reported at the 10, 5, and 1 percent levels

Similarly, in Table A.5, we also test whether educational attainment and employment status are jointly important. We consider whether employed wives of different levels of education entered into new marriages or divorces. For new marriages, the composition shifts towards employed wives with higher levels of education. For divorces, divorces decline the most for employed wives with a middle school level of education but not the lowest level of education. However, the results may be partially due to population composition because the same pattern by education emerges for unemployed wives in Figure A.6. These results suggest that education is more important than employment in the marriage/divorce decision.

Second, in Panel B of Table 4, we consider whether the composition of same-sex marriages changed during the pandemic. While there is a positive coefficient, the impact of the pandemic on new marriages (and divorces) to same-sex couples is statistically insignificant.

Third, we show the impact on who initiates the divorce. There is a clear decline in wives initiating divorce during the pandemic, with a sharp increase in both spouses initiating. A potential explanation for the increase in both spouses initiating divorce is more use of administrative divorce during the pandemic. Administrative divorce requires both spouses to consent to the divorce, there must be no children requiring support, and the couple must have a separate property regime. This is in line with the theoretical prediction of Becker (1973, 1974), suggesting that divorce and the duration of marriage are related to specific investments made during the marriage in the form of children, attachments, and assets. In the absence of these, the opportunity cost of leaving a marriage is potentially minimal, thus explaining the inclination towards administrative divorces. In Panel C, the results align with expectation, where administrative divorces increase during the pandemic while judicial divorces decrease.

Finally, we show the results by length of the marriage, and whether a minor child is present in the household in Panel D. The results in Panel D show that divorce shifts towards newer unions (<10 years) and away from longer marriages (>20 years) during the pandemic. This finding, along with observed increases in unemployment, can be linked with prior evidence suggesting countercyclical divorce/separation probabilities, e.g., unemployment increases the risk of a marriage ending for couples in years 6–10 of marriage (Arkes & Shen, 2014). These findings by marriage duration confirm that “the incentive to separate tend to decline with duration” due to investments made during marriage (Becker, 1974, pg. 23). The results by marriage length add to the age-specific results, suggesting that divorces shift towards younger couples. These younger couples, with shorter marriage durations, have lower marital investments relative to older couples.

### State-level characteristics

We conclude by testing heterogeneous effects at the state level. Table A.7 considers whether states with higher levels of employment, educational attainment, COVID-19 rates, or Human Development Index (HDI) experienced more substantial changes in marriage and divorce rates. We classify a state as having high levels of observable characteristics if the state is above the average level. Table A.7 reveals only two significant heterogeneous effects. First, in high HDI states, marriage rates decline by slightly less. Second, in states with high COVID-19 case rates as of June 30th, 2020, the divorce rate drops by more, suggesting that couples may have reacted to the intensity of the pandemic in their divorce decisions.

## Conclusion

In this study, we consider the initial impact of the COVID-19 pandemic on marriage and divorce rates. Our difference-in-differences results suggest that over March through December of 2020, marriage rates declined by 54% and divorce rates by 43%. Marriage and divorce rates in Mexico also display a notable dynamic effect. During the stay-at-home order, marriage and divorce rates decline by 90–98% but begin to recover immediately afterward. Divorce rates return to baseline levels by September 2020, indicating a full recovery in marital dissolutions. By contrast, while the marriage rate partially recovers, marriage rates remain 30% below baseline levels at the end of 2020, indicating that family formation may be delayed or even permanently prevented during the COVID-19 pandemic. However, our data do not allow us to determine whether marriage rates will fully recover after the initial phase of the pandemic (in 2020). Marriage rates may have fully recovered after vaccines became widely available in Mexico, or the pandemic-related fear subsided.

Then, we consider the impact of the pandemic on divorce (and marriage) characteristics as a percent of all divorces (and marriages). During the pandemic, marriages and divorces skew younger (as a percent), with the youngest individuals least likely to defer marriage and divorce plans. Divorces are also more likely to occur to an employed wife and less likely to be initiated by the wife. Instead, divorces are more likely to be filed jointly during the pandemic, reflecting a shift toward administrative divorce (in percentage terms), which circumvents a lengthy judicial process.

Our results align with existing studies documenting the impact of the pandemic on marriage and divorce rates (Fallesen, 2021, Manning & Payne, 2021, Wagner et al., 2020), though our estimates suggest that the reduction in new marriages and divorces may be more substantial in the middle-income setting as compared to high-income counterparts. These larger reductions in new marriages and new divorces are potentially explained by limited access to vaccines (perpetuating isolation), the lower capacity of the Mexican government to offer a safety net, as well as limited online infrastructure for administrative processes. Further, our results suggest a notable change in couple characteristics. New divorces shift towards the least educated while new marriages shift towards more educated couples during the pandemic.

These findings also leave open questions for future research. First, we cannot say what will happen to marriage and divorce rates past 2020. An important question is whether 2021 and 2022 will bring a boom of new marriages and divorces. Second, while we unpack the characteristics of states and individuals experiencing the change in divorce and marriage rates, we do little to formally consider why marriage and divorce rates changed during the pandemic. Future work could examine the specific mechanisms behind the observed decline in marriage and divorce rates. Finally, our findings do not directly speak to assortativeness or inequality. While we show that the educational composition of new marriages and divorces shifted during the beginning of the pandemic, our results do little to examine how this compositional adjustment affects between-household inequality. Instead, future research could consider whether these compositional adjustments exacerbate societal inequality and marital assortativeness. We leave these open questions for future work.

## Supplementary Information

Supplementary Information

## Footnotes

1 Existing economic literature had initially termed marriage and fertility outcomes as pro-cyclical, i.e., they tend to decline in a poor economy (González-Val & Marcén, 2018, Kondo, 2016, Schaller, 2013). Formal empirical evidence on divorce rates being pro-cyclical, which followed later, are in Hellerstein and Morrill (2011), Schaller (2013). Some contrasting evidence on divorce rates being counter-cyclical is presented in Fischer and Liefbroer (2006), González-Val and Marcén (2018), South and Lloyd (1995). However, evidence for divorce being pro-cyclical is stronger; as noted in Hellerstein and Morrill (2011), both South and Lloyd (1995) and Fischer and Liefbroer (2006) present analyses which do not control for “secular changes in the divorce rate and the economy” which might have led to spurious correlation.

2 Some studies have shown that natural disasters increase marriage and divorce rates (Cicatiello, et al., 2019); and a decrease in marital age (Khanna & Kochhar, 2020) as aftereffects. By contrast, there is also evidence of a decrease in marriage and divorce rates following natural and man-made disasters (Ahmed, 2017, Cohan et al., 2009). See the literature review in Section 2 for a more thorough description of these findings.

3 The legal age of marriage in Mexico was changed to 18 years in 2019 by Federal reform, before that, the legal age of marriage was 14 years for women and 16 years for men (de Gobernación, 2019). Our choice of the age range for the analysis is informed by this change in the legal age of marriage enacted by the 2019 reform to the Federal Civil Code.

4 Because marriage and divorce rates exhibit seasonal trends, we account for seasonality using indicators for each month of the year.

5 This strategy follows similar papers in the literature that use year-over-year differences-in-differences, including Bullinger et al. (2020), Leslie and Wilson (2020), Hoehn-Velasco, Silverio-Murillo and de la Miyar (2021a), and de la Miyar, Hoehn-Velasco and Silverio-Murillo (2021). We also present this empirical strategy visually in Figure A.1.

6 Using a different methodology, Ghaznavi et al. (2022) had similar findings.

7 Manning and Payne (2021), using data from Arizona, Florida, Missouri, New Hampshire, and Oregon, find that after the initial decline, marriages for Arizona and New Hampshire and divorces for Arizona recover to pre-pandemic baseline levels. Wagner et al. (2020) documents a 26–44% decrease in marriages and a 15% decrease in marriage applications, varying by states and metropolitan statistical areas (MSAs) considered in the United States.

8 First, Mexico’s government was the only provider of vaccines and struggled to mobilize a vaccine response. Vaccination against COVID-19 was not available until the spring of 2021. Second, Mexico’s government offered almost no safety net during the initial phases of the pandemic. For example, IMF (2021)’s estimates indicate that the fiscal stimulus provided in Mexico (0.7% of its GDP) was substantially lower than the aid provided in the United States (25%) or Japan (50%). Finally, Mexico’s government has limited administrative capacity, particularly e-government development. Mexico’s online infrastructure for administrative processes is far behind high-income countries, which could explain the larger decline in the execution of new marriages and new divorces (e.g., Mexico ranked 61 in e-government development in 2020 (UN, 2020)).

9 The literature has demonstrated that the initial COVID-19 pandemic significantly impacted women relative to men (Albanesi & Kim, 2021, Alon, Doepke, Olmstead-Rumsey & Tertilt, 2020a, b, Croda & Grossbard, 2021, Pitts, 2021). The pandemic particularly impacted women due to both the service sector-specific impacts of the pandemic (Alon et al., 2020b) and the higher childcare burdens due to school and daycare closures (Alon et al., 2020b, Bansak & Starr, 2021, Croda & Grossbard, 2021, Heggeness, 2020, Yamamura & Tsustsui, 2021, Zamarro & Prados, 2021).

10 Indeed, when men’s average income decline, a lower demand for single women’s work in marital production is expected (Grossbard, 2005). In addition, a woman’s marital *reservation wage* is not only affected by her own income but by the income of other women. If other women are doing poorly in the economy, then the marital reservation wage is low (Grossbard, 2005).

11 Schaller (2013) provide suggestive evidence on the permanence of the effect of an unemployment shock on marriage.

12 Most of these early-age marriages observed in Das and Dasgupta (2020), Ebitt (2015), Khanna and Kochhar (2020) involve dowries, which can be thought of as a consumption smoothing step for the dowry-receiving household to alleviate the financial loss induced by the disaster.

13 See Balmori de la Miyar, Hoehn-Velasco and Silverio-Murillo (2020), de la Miyar et al. (2021), Hoehn-Velasco et al. (2021a, 2022), Silverio-Murillo, Hoehn-Velasco, de la Miyar and Méndez (2021).

14 To illustrate the sequence of events related to the pandemic, we outline the specific timeline of notable events in Table A.1.

15 For new marriages, all marriage filings must be performed at a state registrar, according to local legislation. In divorces, registrars and courts play different roles depending on the divorce type. In administrative divorce cases, all parties agree to the dissolution terms, and the filing is done at state registrars. Conversely, in the case of judicial divorces, a civil lawsuit must be brought to a state court, which subpoenas both parties.

16 These states include Aguascalientes (April–May), Baja California (April–July), Baja California Sur (April–May), Campeche (March–May), Chihuahua (March-April), Mexico City (March–June), Guanajuato (March–June), Hidalgo (March–June), Morelos (March–July), San Luis Potosi (March-June), Sonora (March–June), Tamaulipas (March–May), Tlaxcala (March–July), and Yucatan (March–June).

17 Before the amendment, marriage was allowed from 16 years for men and from 14 years for women (de Gobernación, 2019).

18 For more on changes in divorce laws see Lew and Beleche (2008); for more on no-fault unilateral divorce in Mexico see Murillo (2019), Hoehn-Velasco and Silverio-Murillo (2020), García-Ramos (2021), Hoehn-Velasco and Penglase (2021b), and Hoehn-Velasco and Penglase (2021c).

19 In addition to these generational trends, we also show age-specific rates in Figure A.3.

20 While this comparison is imperfect. Our main analysis considers the pre-pandemic months in 2020. Thus, our analysis considers the pre-post change in 2020 relative to similar months of earlier years.

21 Hoehn-Velasco and Penglase (2021c) also outlines the specific causes for divorce and the full data description.

22 Still, in the robustness checks, we test the divorce registration date instead of the execution date.

23 We use the population over 15 as the INEGI reports the quarterly working-age population, which includes those 15 and over. We prefer 15 and over to the total population, as it more closely represents the marriageable age population. This age range is also used in similar papers (Hoehn-Velasco & Silverio-Murillo, 2020, Hoehn-Velasco & Penglase, 2021c).

24 This is also reflected in Figure A.4, where the divorce and marriage rates by the wife’s age are shown over both years. In Panel A, marriages clearly are most common among the 15–29 group, which continues in 2020, but the magnitude substantially falls. In Panel B, divorces are most common among those 30–44, though all rates fall by roughly two-thirds in 2020Q2 versus 2019Q2.

25 Education at the primary level is defined as primary education or less. Higher education is defined as a college or technical education.

26 In addition to these main summary statistics, we also show additional features of marriages and divorces in Table A.2. Here the summary statistics suggest a notable shift towards marriages where the wife is employed. New divorces and marriages are also composed of a larger percentage of same-sex couples.

27 Note the percentages do not sum, as there are missing values in the microdata.

28 This is a necessary assumption to establish causality when using the difference-in-differences technique.
