# Supplementary material for: Marriage and divorce during a pandemic: the impact of the COVID-19 pandemic on marital formation and dissolution in Mexico
Source: Rev Econ Househ. 2023 Apr 11:1–32. Online ahead of print. doi: 10.1007/s11150-023-09652-y (PMC10088673; doi:10.1007/s11150-023-09652-y)
Supplement: Supplementary file 1 — Supplementary Information [file 11150_2023_9652_MOESM1_ESM.pdf]

A Appendix-For Online Publication

A.1 Additional Tables

Table A.1: Timeline of Mexico's Initial Pandemic and National Lockdown

February 28th	First COVID-19 Case in Mexico City
March 11th	WHO Declares Pandemic
March 15th	Schools Closed
March 18th	Some State Registrars and State Courts Closed
March 23rd	Stay-at-Home Order Issued
April 19th	Some State Courts Partially Reopen
May 30th	National Lockdown Ends
June 8th	Some State Courts Integrate Digitial Reopening

Table A.2: Summary Statistics: Additional Outcomes

	**Pre-Post Pandemic**				
	Pre-COVID		Post-COVID		Pre-Post Pandemic
	Mean	Std. Dev.	Mean	Std. Dev.	Diff.
**Overall**					
Divorces	401.6	373.1	214.4	270.1	-187.3***
Marriages	1,353.6	1,201.9	701.3	800.4	-652.3***
**% Marriages Same Educ.**					
Both Primary	6.1	4.1	4.5	4.1	-1.5***
Both Middle	14.2	4.1	11.4	4.5	-2.8***
Both Secondary	13.8	4.6	14.6	5.2	0.8**
Both Higher-Ed	19.1	6.5	23.3	8.4	4.2***
**% Divorces Same Educ.**					
Both Primary	5.2	4.9	6.3	13.7	1.1
Both Middle	10.9	5.2	11.0	14.2	0.1
Both Secondary	9.0	5.0	8.4	6.9	-0.6
Both Higher-Ed	12.6	6.6	12.6	11.7	-0.1
**% Marriages Employ.**					
Wife Employed	51.6	12.3	61.3	17.7	9.7***
Husband Employed	96.6	1.5	96.2	2.5	-0.4**
Both Employed	47.4	10.8	51.2	12.1	3.9***
**% Divorces Employ.**					
Wife Employed	66.5	9.3	69.3	18.3	2.7**
Husband Employed	94.2	3.8	94.2	8.1	-0.1
Both Employed	44.5	15.3	43.8	23.7	-0.7
**% Marriages Char.**					
Same Sex Marriages	0.6	1.1	0.9	1.8	0.3***
**% Divorces Char.**					
Same Sex Divorces	0.1	0.3	0.2	1.0	0.1**
Male Initiated	24.8	10.9	21.9	17.1	-2.9***
Female Initiated	29.5	13.2	24.9	17.1	-4.6***
Both Initiated	45.7	22.5	53.2	31.2	7.5***
Alimony Kids	47.5	13.1	37.9	22.3	-9.6***
Alimony Spouse	4.8	5.2	3.0	5.0	-1.8***
Alimony Kids and Spouse	3.9	4.6	2.5	4.8	-1.4***
Has Child	48.9	11.5	39.1	21.9	-9.7***
Marriage length	15.8	1.1	13.8	5.2	-1.9***
N	1,216		320		1,536

SOURCE: INEGI marriage and divorce microdata.
NOTES: Data aggregated to the national level, observations represent month-year combinations. The percentage outcomes are per 100 marriages or divorces. Significance levels reported at the 10, 5, and 1 percent levels.

Table A.3: Robustness: Divorce and Marriage Rates

Rate Per 1,000:	Marriages					Divorces					
	(1) Controls	(2) Trends	(3) No CDMX	(4) Replace Missing	(5) National	(6) Controls	(7) Trends	(8) No CDMX	(9) Replace Missing	(10) National	(11) Date Registered
1(COVID-19)	-0.24***	-0.24***	-0.23***	-0.24***	-0.24***	-0.06***	-0.06***	-0.06***	-0.06***	-0.06***	-0.03***
	(0.01)	(0.01)	(0.01)	(0.01)	(0.03)	(0.01)	(0.01)	(0.01)	(0.01)	(0.02)	(0.01)
N	1,536	1,536	1,488	1,532	48	1,536	1,536	1,488	1,513	48	1,536
Adjusted R-squared	0.56	0.56	0.55	0.56	0.89	0.79	0.81	0.79	0.80	0.60	0.81
Wild Bootstrap P-Value	0.00	0.00	0.00	0.00	0.01	0.00	0.00	0.00	0.00	0.09	0.00
2019 March-December Mean	0.44	0.44	0.45	0.44	0.44	0.14	0.14	0.14	0.14	0.14	0.12
COVID-19 Percentage Change	-54.3%	-54.4%	-51.6%	-54.1%	-54.3%	-43.4%	-43.4%	-41.0%	-40.2%	-43.4%	-28.0%
Baseline FE	X	X	X	X	X	X	X	X	X	X	X

Source: INEGI marriage and divorce microdata.
Notes: Post-COVID is a dummy variable capturing the impact of the pandemic, which equals one from March 2020 to December 2020. Baseline fixed effects include the state, month, and year. The divorce and marriage rates are reported per 1,000 persons 15 and over. Results weighted by the state population 15 and over. Robust standard errors are clustered at the state level. Significance levels reported at the 10, 5, and 1 percent levels.

Table A.4: Percentage of Marriages and Divorces by Shared Spousal Education

%	Marriages					Divorces				
	(1)	(2)	(3)	(4)	(5)	(6)	(7)	(8)	(9)	(10)
	Same Education Spouses	Both Primary Education	Both Middle School Education	Both Secondary Education	Both College (Or Higher) Education	Same Education Spouses	Both Primary Education	Both Middle School Education	Both Secondary Education	Both College (Or Higher) Education
1(COVID-19)	1.84	0.02	-1.26*	0.74	2.34***	1.81	1.63*	-0.94*	0.54	0.58
	(1.43)	(0.39)	(0.72)	(0.47)	(0.66)	(1.90)	(0.82)	(0.54)	(0.86)	(0.58)
N	1,532	1,532	1,532	1,532	1,532	1,513	1,513	1,513	1,513	1,513
Adjusted R-squared	0.78	0.76	0.67	0.81	0.76	0.89	0.70	0.77	0.74	0.88
Wild Bootstrap P-Value	0.21	0.97	0.09	0.09	0.00	0.42	0.01	0.13	0.54	0.34
2019 March-December Mean	54.23	5.53	14.25	14.73	19.72	39.74	4.30	11.57	9.69	14.16
COVID-19 Percentage Change	3.4%	0.3%	-8.8%	5.0%	11.9%	4.6%	38.0%	-8.2%	5.6%	4.1%
Baseline FE	X	X	X	X	X	X	X	X	X	X

SOURCE: INEGI marriage and divorce microdata.
NOTES: Post-COVID is a dummy variable capturing the impact of the pandemic, which equals one from March 2020 to December 2020. Baseline fixed effects include the state, month, and year. The divorce and marriage rates are reported per 1,000 persons 15 and over. Results weighted by the state population 15 and over. Robust standard errors are clustered at the state level. Significance levels reported at the 10, 5, and 1 percent levels.

Table A.5: Percentage of Marriages and Divorces by the Wife's Education/Employment Combination

%	Marriages				Divorces			
	(1)	(2)	(3)	(4)	(5)	(6)	(7)	(8)
	Wife Employed Primary Education	Wife Employed Middle School Education	Wife Employed Secondary Education	Wife Employed College (Or Higher) Education	Wife Employed Primary Education	Wife Employed Middle School Education	Wife Employed Secondary Education	Wife Employed College (Or Higher) Education
1(COVID-19)	-0.06	-0.51	0.82	2.48***	0.75	-1.77**	-0.33	0.04
	(0.22)	(0.51)	(0.55)	(0.71)	(0.79)	(0.80)	(0.81)	(0.39)
N	1,532	1,532	1,532	1,532	1,513	1,513	1,513	1,513
Adjusted R-squared	0.41	0.74	0.78	0.76	0.58	0.77	0.81	0.86
Wild Bootstrap P-Value	0.85	0.32	0.14	0.00	0.58	0.03	0.82	0.92
2019 March-December Mean	1.93	8.26	13.36	24.57	3.79	12.73	13.83	19.29
COVID-19 Percentage Change	-3.1%	-6.2%	6.1%	10.1%	19.9%	-13.9%	-2.4%	0.2%
Baseline FE	X	X	X	X	X	X	X	X

SOURCE: INEGI marriage and divorce microdata.
NOTES: Post-COVID is a dummy variable capturing the impact of the pandemic, which equals one from March 2020 to December 2020. Baseline fixed effects include the state, month, and year. The divorce and marriage rates are reported per 1,000 persons 15 and over. Results weighted by the state population 15 and over. Robust standard errors are clustered at the state level. Significance levels reported at the 10, 5, and 1 percent levels.

Table A.6: Percentage of Marriages and Divorces by the Wife's Education/Unemployment Combination

%	Marriages				Divorces			
	(1)	(2)	(3)	(4)	(5)	(6)	(7)	(8)
	Wife Unemployed Primary Education	Wife Unemployed Middle School Education	Wife Unemployed Secondary Education	Wife Unemployed College (Or Higher) Education	Wife Unemployed Primary Education	Wife Unemployed Middle School Education	Wife Unemployed Secondary Education	Wife Unemployed College (Or Higher) Education
1(COVID-19)	-0.39	-1.84**	-0.38	0.33*	0.05	-1.43**	-0.36	-0.34*
	(0.49)	(0.68)	(0.41)	(0.16)	(0.26)	(0.57)	(0.64)	(0.18)
N	1,532	1,532	1,532	1,532	1,513	1,513	1,513	1,513
Adjusted R-squared	0.78	0.71	0.77	0.73	0.75	0.75	0.64	0.75
Wild Bootstrap P-Value	0.47	0.02	0.36	0.08	0.85	0.02	0.61	0.08
2019 March-December Mean	7.44	16.44	13.16	3.73	4.53	9.64	5.51	4.06
COVID-19 Percentage Change	-5.2%	-11.2%	-2.9%	8.8%	1.1%	-14.9%	-6.5%	-8.5%
Baseline FE	X	X	X	X	X	X	X	X

SOURCE: INEGI marriage and divorce microdata.
NOTES: Post-COVID is a dummy variable capturing the impact of the pandemic, which equals one from March 2020 to December 2020. Baseline fixed effects include the state, month, and year. The divorce and marriage rates are reported per 1,000 persons 15 and over. Results weighted by the state population 15 and over. Robust standard errors are clustered at the state level. Significance levels reported at the 10, 5, and 1 percent levels.

Table A.7: Heterogeneous Effects: Divorce and Marriage Rates

	Marriage Rate	Divorce Rate
	(1)	(2)
1(COVID-19) x 1(High Working in 2020Q3)	-0.028	0.017
	(0.027)	(0.014)
1(COVID-19)	-0.226***	-0.069***
	(0.016)	(0.014)
1(COVID-19) x 1(High Work Hours in 2020Q3)	0.043*	0.007
	(0.024)	(0.015)
1(COVID-19)	-0.263***	-0.065***
	(0.016)	(0.014)
1(COVID-19) x 1(High Unemployed in 2020Q3)	0.032	-0.010
	(0.026)	(0.014)
1(COVID-19)	-0.255***	-0.056***
	(0.020)	(0.011)
1(COVID-19) x 1(High Female-to-Male Ratio)	-0.022	0.024
	(0.028)	(0.018)
1(COVID-19)	-0.225***	-0.076***
	(0.024)	(0.016)
1(COVID-19) x 1(High Share College Educated)	0.024	-0.019
	(0.026)	(0.016)
1(COVID-19)	-0.248***	-0.053***
	(0.017)	(0.008)
1(COVID-19) x 1(High Share Married)	-0.002	-0.003
	(0.027)	(0.015)
1(COVID-19)	-0.238***	-0.059***
	(0.022)	(0.013)
1(COVID-19) x 1(High HDI)	0.052**	-0.020
	(0.026)	(0.013)
1(COVID-19)	-0.268***	-0.050***
	(0.023)	(0.011)
1(COVID-19) x 1(High COVID-19 Rate June 2020)	-0.004	-0.026**
	(0.026)	(0.013)
1(COVID-19)	-0.238***	-0.050***
	(0.019)	(0.011)
Observations	1,536	1,536
Pre-Lockdown Mean Dependent	0.59	0.13
Baseline FE	X	X
Time Trends	X	X

SOURCE: INEGI marriage and divorce microdata.
NOTES: Post-COVID is a dummy variable capturing the impact of the pandemic, which equals one from March 2020 to December 2020. Baseline fixed effects include the state, month, and year. The divorce and marriage rates are reported per 1,000 persons 15 and over. Results weighted by the state population 15 and over. Robust standard errors are clustered at the state level. Significance levels reported at the 10, 5, and 1 percent levels.

A.2 Additional Figures

Figure A.1: COVID-19 Difference-in-differences Methodology

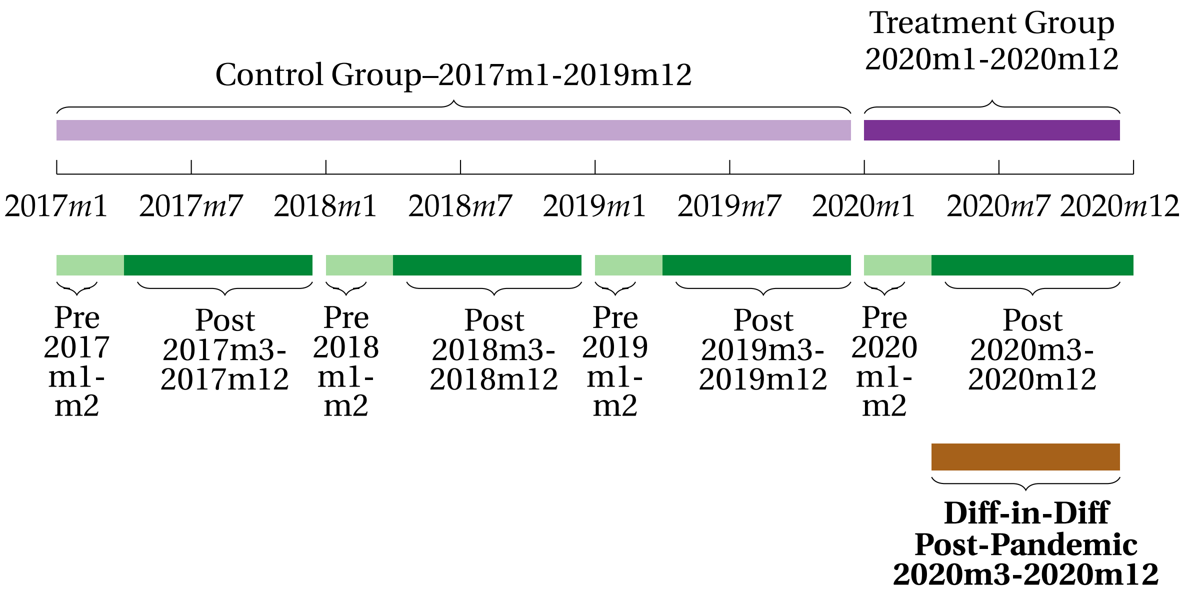

Figure A.2: Map of State Registrars and Courts Closed During the Lockdown

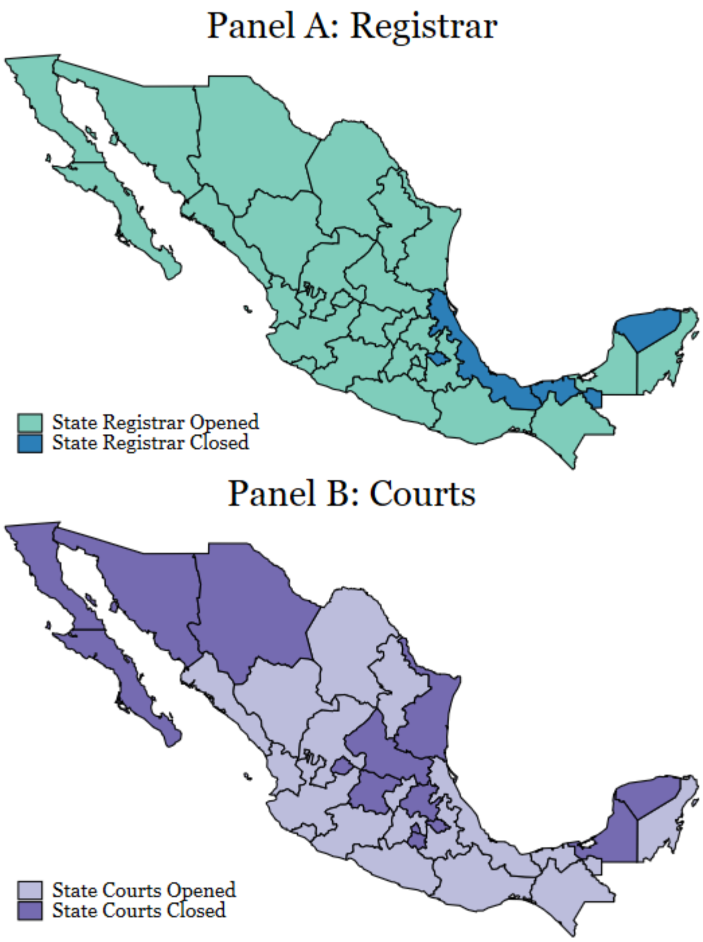

SOURCE: State Registrars press releases, State Courts press releases, and author's phone inquires to State Registrars.

Figure A.3: Monthly Pattern of Marriage and Divorce Rates

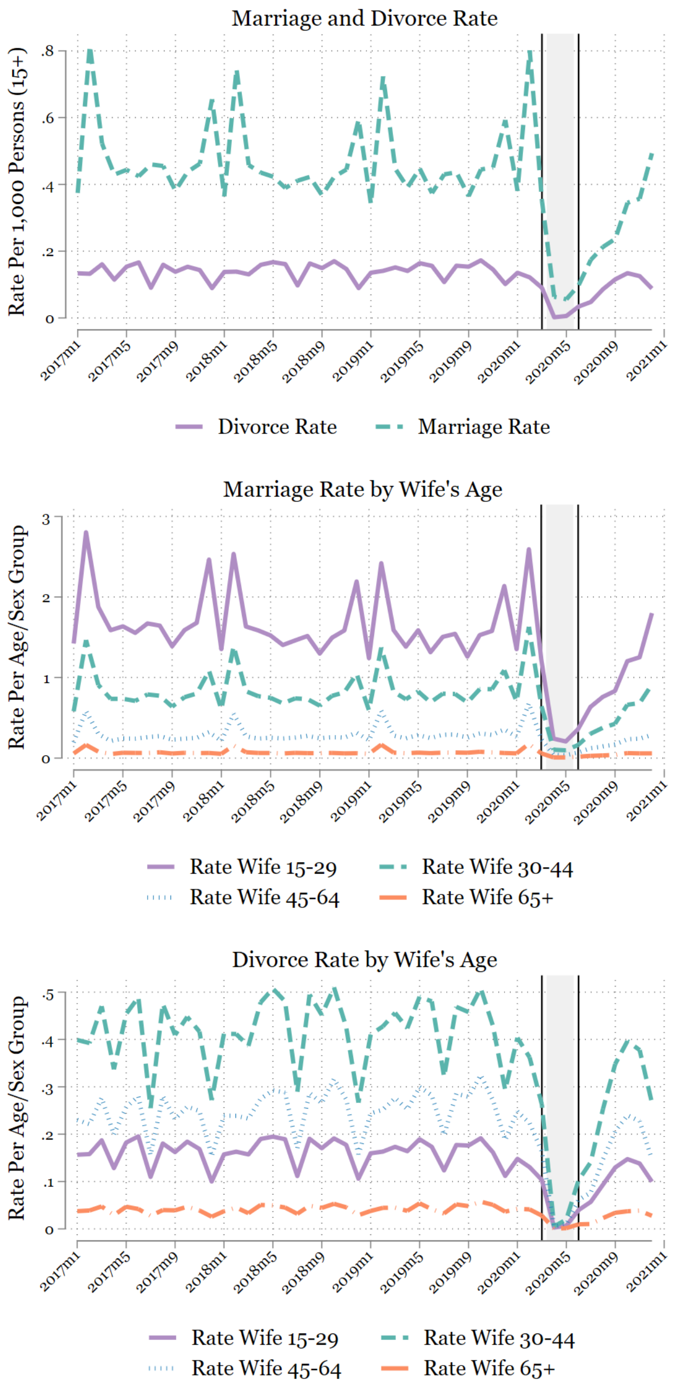

SOURCE: INEGI marriage and divorce microdata.
NOTES: Data aggregated to the national level, observations represent month-year combinations. The divorce and marriage rates are reported per 1,000 persons 15 and over. Age-and-sex-specific rates are per 1,000 persons of that population.

Figure A.4: Background: Marriage and Divorce Rates, 2020Q2 v. 2019Q2

Panel A: Age of Wife in Marriages

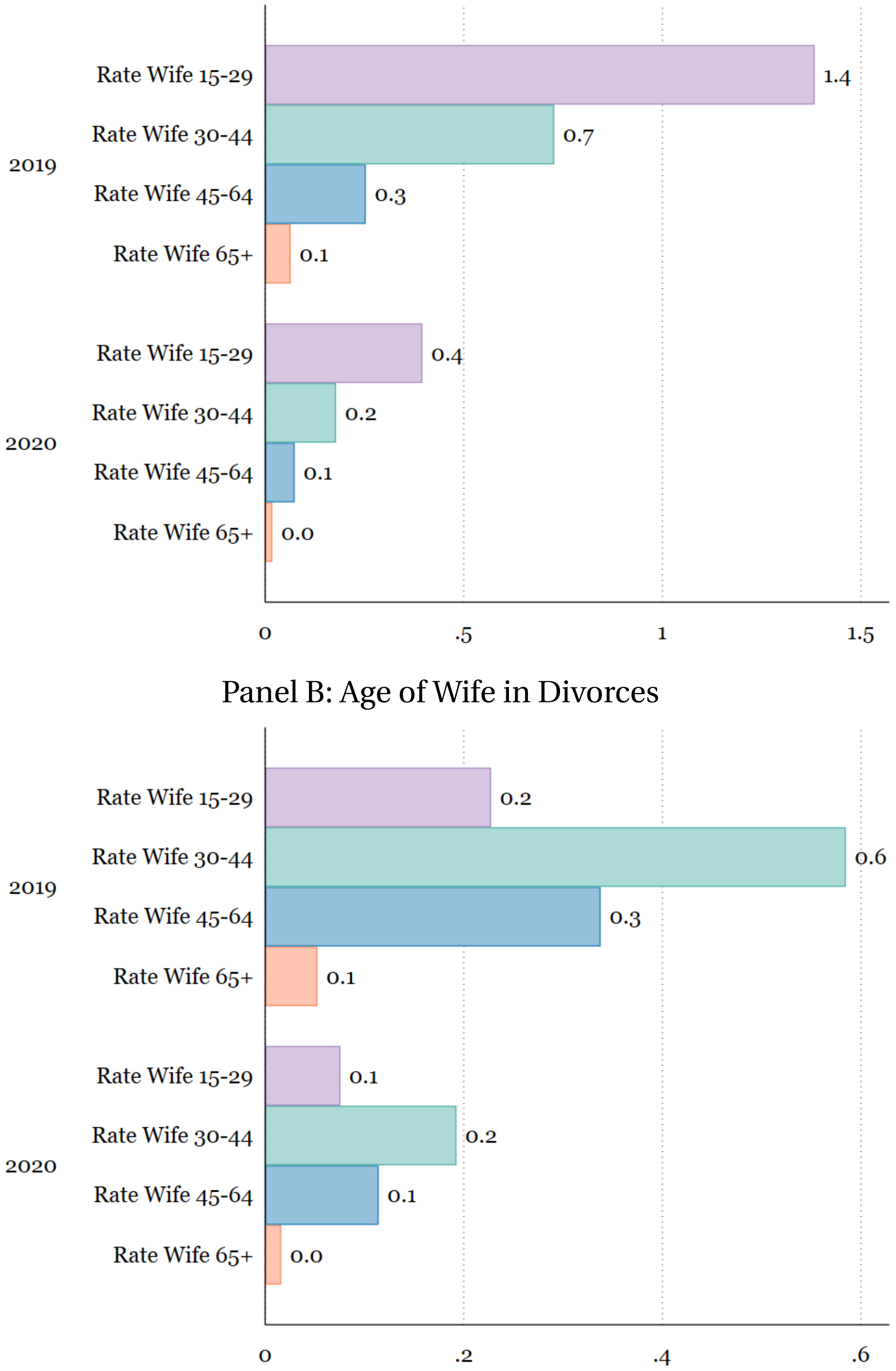

SOURCE: INEGI marriage and divorce microdata.

Figure A.5: Event Study Results: Log of Marriage and Divorce Rates

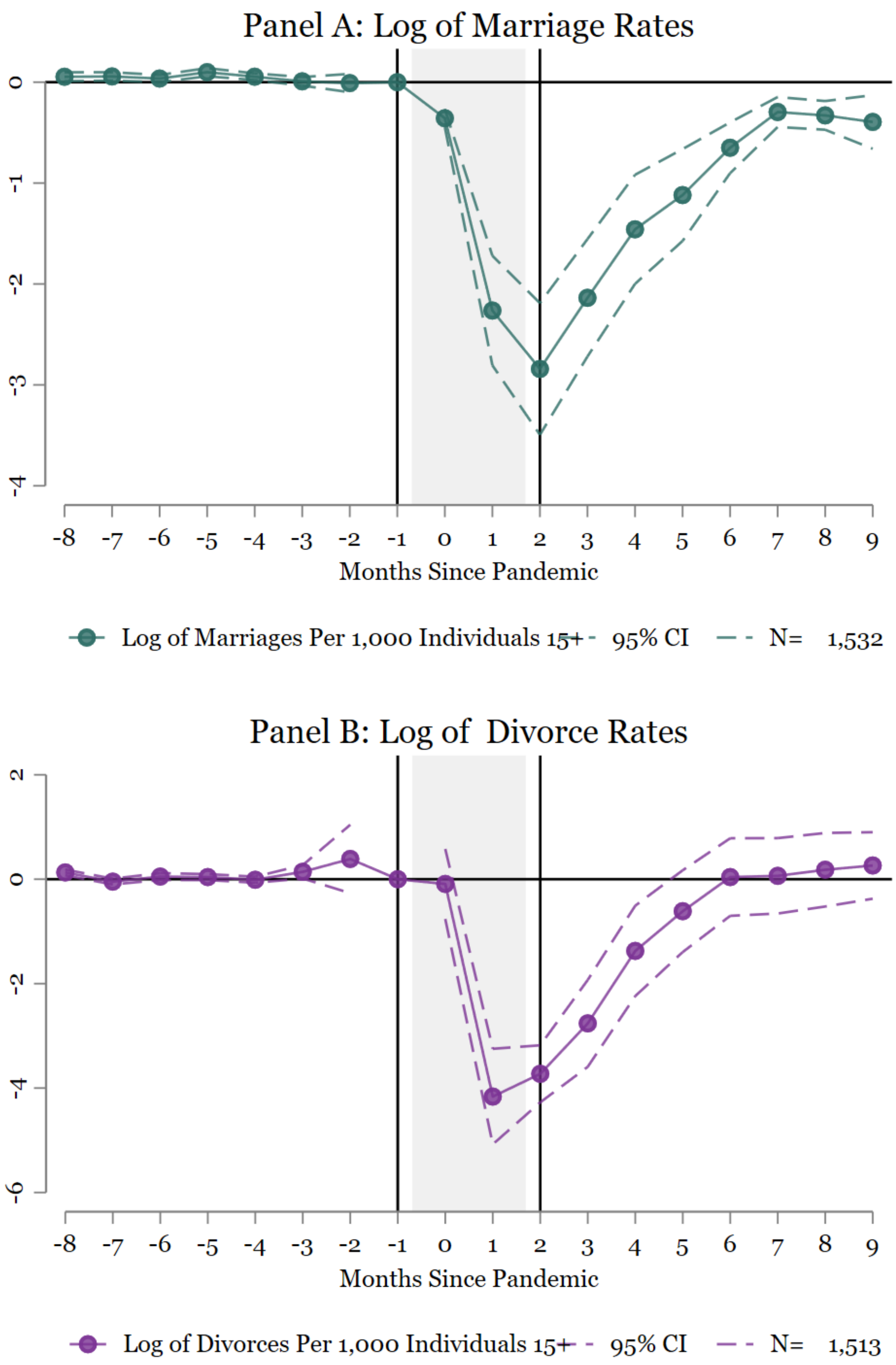

Source: INEGI marriage and divorce microdata.
Notes: Plotted coefficients are event-study dummy variables, β_q. Each plotted point represents the number of months before and after the start of the pandemic. The event study considers 2017-2020, with 2020m2 as the omitted period. Solid lines represent point estimates. Dotted lines display the 95 percent confidence intervals. Baseline fixed effects include the state, month, and year. The divorce and marriage rates are reported per 1,000 persons 15 and over. Robust standard errors are clustered at the state level.

Figure A.6: Event Study Results: Marriage and Divorce Rates by Type

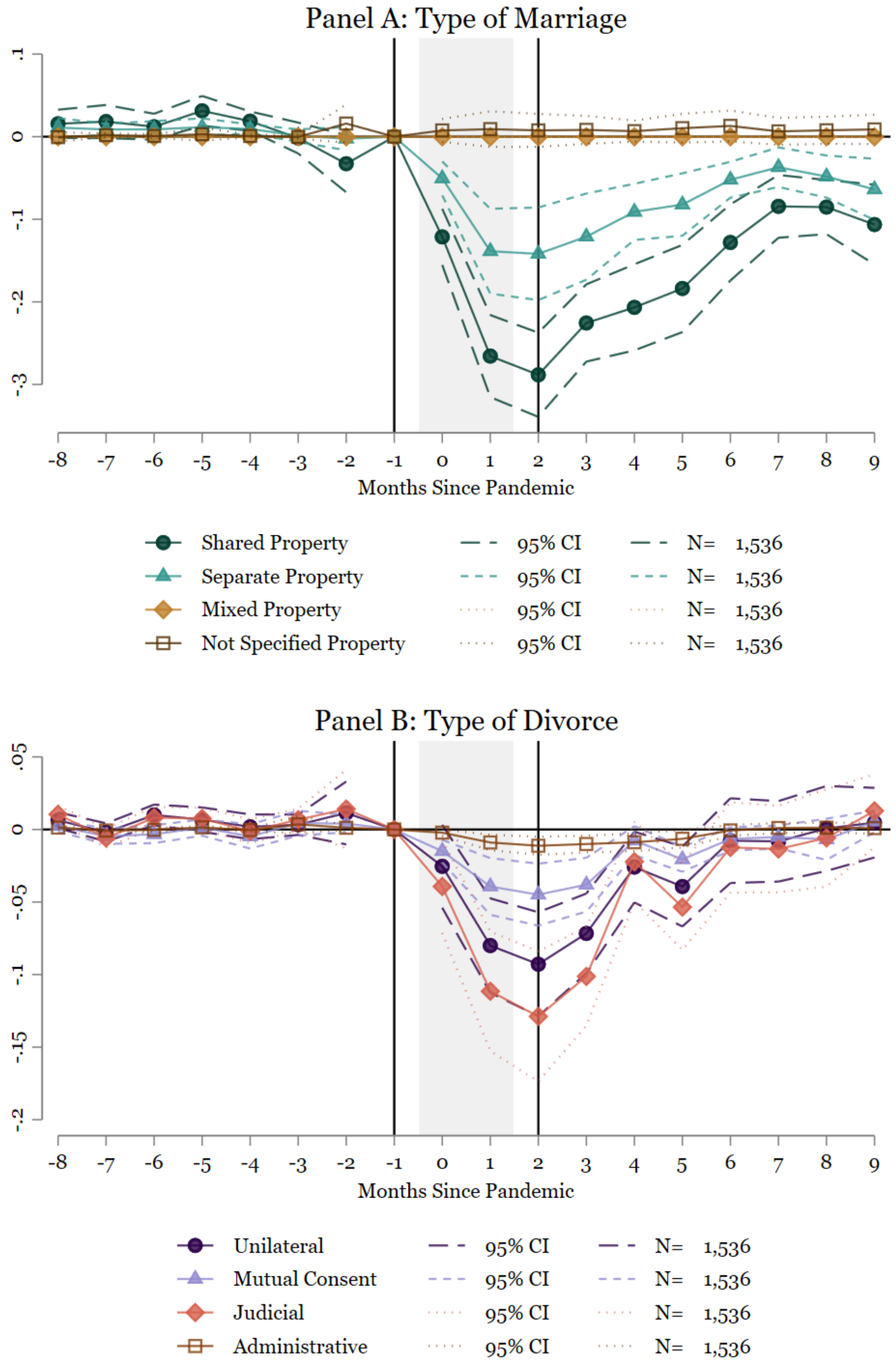

SOURCE: INEGI marriage and divorce microdata.
NOTES: Plotted coefficients are event-study dummy variables, β_q. Each plotted point represents the number of months before and after the start of the pandemic. The event study considers 2017-2020, with 2020m2 as the omitted period. Solid lines represent point estimates. Dotted lines display the 95 percent confidence intervals. Baseline fixed effects include the state, month, and year. The divorce and marriage rates are reported per 1,000 persons 15 and over. Robust standard errors are clustered at the state level.
